# HIV-1 and M-PMV RNA Nuclear Export Elements Program Viral Genomes for Distinct Cytoplasmic Trafficking Behaviors

**DOI:** 10.1371/journal.ppat.1005565

**Published:** 2016-04-12

**Authors:** Ginger M. Pocock, Jordan T. Becker, Chad M. Swanson, Paul Ahlquist, Nathan M. Sherer

**Affiliations:** 1 McArdle Laboratory for Cancer Research and Institute for Molecular Virology, University of Wisconsin-Madison, Madison, Wisconsin, United States of America; 2 Morgridge Institute for Research, University of Wisconsin-Madison, Madison, Wisconsin, United States of America; 3 Department of Infectious Diseases, King’s College London, London, United Kingdom; 4 Howard Hughes Medical Institute, University of Wisconsin-Madison, Madison, Wisconsin, United States of America; Duke University Medical Center, UNITED STATES

## Abstract

Retroviruses encode *cis*-acting RNA nuclear export elements that override nuclear retention of intron-containing viral mRNAs including the full-length, unspliced genomic RNAs (gRNAs) packaged into assembling virions. The HIV-1 Rev-response element (RRE) recruits the cellular nuclear export receptor CRM1 (also known as exportin-1/XPO1) using the viral protein Rev, while simple retroviruses encode constitutive transport elements (CTEs) that directly recruit components of the NXF1(Tap)/NXT1(p15) mRNA nuclear export machinery. How gRNA nuclear export is linked to trafficking machineries in the cytoplasm upstream of virus particle assembly is unknown. Here we used long-term (>24 h), multicolor live cell imaging to directly visualize HIV-1 gRNA nuclear export, translation, cytoplasmic trafficking, and virus particle production in single cells. We show that the HIV-1 RRE regulates unique, *en masse*, Rev- and CRM1-dependent “burst-like” transitions of mRNAs from the nucleus to flood the cytoplasm in a non-localized fashion. By contrast, the CTE derived from Mason-Pfizer monkey virus (M-PMV) links gRNAs to microtubules in the cytoplasm, driving them to cluster markedly to the centrosome that forms the pericentriolar core of the microtubule-organizing center (MTOC). Adding each export element to selected heterologous mRNAs was sufficient to confer each distinct export behavior, as was directing Rev/CRM1 or NXF1/NXT1 transport modules to mRNAs using a site-specific RNA tethering strategy. Moreover, multiple CTEs per transcript enhanced MTOC targeting, suggesting that a cooperative mechanism links NXF1/NXT1 to microtubules. Combined, these results reveal striking, unexpected features of retroviral gRNA nucleocytoplasmic transport and demonstrate roles for mRNA export elements that extend beyond nuclear pores to impact gRNA distribution in the cytoplasm.

## Author Summary

How cellular and viral mRNAs are trafficked inside the cell is poorly understood. In this study, we use fluorescence microscopy to visualize HIV-1 mRNA nuclear export, translation, trafficking, and virus particle assembly in single living cells, focusing on the full-length viral genomic RNAs (gRNAs) that are packaged into virus particles. We demonstrate that HIV-1 gRNAs exhibit dramatic “burst”-like transitions from the nucleus to fill the cytoplasm prior to the onset of virus particle assembly. This process is regulated by the viral Rev protein and the cellular nuclear export factor CRM1. By contrast, Mason-Pfizer monkey virus (M-PMV), a pathogenic non-human retrovirus that does not exploit CRM1, links gRNAs to the microtubule cytoskeleton in a mechanism regulated by an alternative cellular nuclear export factor, NXF1. Combined, our comparative studies reveal two unanticipated mRNA nucleocytoplasmic transport behaviors programmed by contrasting mRNA nuclear export factors. Further elucidation of the molecular details underpinning these unique trafficking behaviors may inform the development of antiviral strategies targeting viral gene expression and/or infectious virion assembly.

## Introduction

Human immunodeficiency virus type 1 (HIV-1) replication requires a tightly orchestrated series of post-transcriptional regulatory events encompassing the nuclear export, translation and packaging of viral full-length positive sense genomic RNAs (gRNAs) [1–3]. Viral gRNAs are transcribed in the nucleus as pre-mRNAs by cellular RNA polymerase II, 5’ 7mG capped and 3’ polyadenylated. The bulk of these transcripts undergo complex alternative splicing to generate the viral messenger RNAs (mRNAs). However, a subset of unspliced gRNAs are exported from the nucleus, introns intact, to serve both as viral mRNAs encoding the structural polyproteins Gag and Gag-Pol as well as the core genetic substrate (*i*.*e*., the genome) packaged as a dimer into virions assembling at the plasma membrane [4–6].

The nuclear export of gRNAs is mediated by the viral auxiliary protein Rev that engages a structured *cis-*acting RNA signal, the Rev response element (RRE), found in unspliced gRNAs and partially spliced viral transcripts that encode the viral proteins Vif, Vpr, Vpu, and Envelope (Env) [7–11]. Rev multimerizes on the RRE and binds the cellular factor chromosome region maintenance 1 (CRM1, also known as exportin-1 or XPO-1) to form a viral ribonucleoprotein (vRNP) transport complex capable of exporting intron-containing transcripts from the nucleus to the cytoplasm [12–16]. In the cytoplasm, gRNAs disengage from Rev and CRM1 and are translated to generate robust levels of Gag and Gag-Pol despite lacking an exon-junction complex (EJC) that, for spliced mRNAs, promotes translation [17,18]. Therefore, gRNAs necessarily recruit viral and cellular factors that help to maintain gRNA stability and facilitate translation [2,19]. Also, the virus must regulate a switch in the cytoplasm to terminate Gag/Gag-Pol translation and initiate gRNA packaging [6,20]. How these activities involving Rev, gRNAs, Gag, and cellular factors are coordinated spatially and temporally is poorly understood.

Many retroviruses lack Rev equivalent proteins and do not exploit CRM1, instead encoding RNA constitutive transport elements (CTEs) that directly recruit components of the NXF1(TAP)/NXT1(p15) mRNA nuclear export machinery [5,21–24]. The best studied CTE is derived from Mason-Pfizer monkey virus (M-PMV), a betaretrovirus and prototype for D-type virion assembly, wherein viral capsids form in the cytoplasm prior to their transport to the plasma membrane for budding and release [25,26]. This pathway differs from the C-type pathway used by HIV-1, wherein capsids assemble in direct association with the plasma membrane [27,28]. Depending on the context, one or more copies of M-PMV’s CTE is sufficient to render HIV-derived intron-bearing viral transcripts Rev- and CRM1-independent [24,29,30]. Functionally equivalent RNA structural elements have been identified in other retroviruses [31–36], endogenous retroelements [37–40], and even a subset of cellular mRNAs [41,42]. Notably, a CTE with remarkable structural and sequence similarity to that of M-PMV was recently identified in an intron-retaining variant of the *NXF1* mRNA itself [41,43]. Thus, the M-PMV CTE likely resulted from co-option of an existing mechanism of cellular gene regulation.

CRM1 mediates the nuclear export of proteins encoding nuclear export signal (NES) peptides and a small subset of cellular RNAs including 5S ribosomal RNAs, U snRNAs, and select mRNAs regulated by NES-bearing adaptor proteins [21,22,44–46]. By contrast, NXF1/NXT1 regulates the bulk of cellular mRNA nuclear export through binding to RNA-binding proteins including ALY/REF, SR family proteins, and components of transcription export complexes 1 and 2 (TREX1/2) [45–49]. The CTE’s capacity to recruit NXF1/NXT1 and associated co-factors (*e*.*g*., SR family proteins) targets intron-retaining transcripts to polyribosomes, thereby ensuring cytoplasmic utilization despite the lack of splicing and an exon junction complex [31,41,50–53]. Thus, why HIV-1 and other genetically complex retroviruses evolved to express a co-factor (*e*.*g*., Rev) to exploit CRM1 instead of the NXF1/NXT1 pathway is unclear. Indeed, replacing HIV-1’s RRE with one or more CTEs is sufficient to support viral replication, albeit with reduced efficiency [54,55]. Similarly, replacement of the CTE in M-PMV with the HIV-1 RRE expressed with Rev supports infectious M-PMV production but to suboptimal levels [56]. On the other hand, Rev (and by proxy, CRM1) have been implicated in other stages of the HIV-1 life cycle including viral pre-mRNA processing [57,58], Gag translation [5,59–62], gRNA packaging into virions [63,64], and the efficiency of virus particle assembly [3,30,65–67].

A potential explanation for the above dichotomy is that the Rev/CRM1 or NXF1/NXT1 export machineries confer distinct cytoplasmic trafficking outcomes on retroviral gRNAs, *i*.*e*., governing differential nucleocytoplasmic transport dynamics and/or subcellular localization relevant to aspects of the productive phase. However, these processes have never been directly visualized. Here, we use live cell imaging to show that the Rev/RRE/CRM1 pathway regulates dramatic *en masse* gRNA nuclear export events, with RRE-bearing HIV-1 gRNA transcripts dispersing throughout the cytoplasm in conjunction with virus particle assembly. By contrast, reprogramming otherwise identical HIV-1 gRNAs to be Rev/CRM1-independent by replacing the RRE with multiple copies of the CTE derived from M-PMV resulted in transcripts coalescing at the nuclear membrane and clustering to the centrosome that forms the core of the microtubule-organizing center (MTOC). Analogous *en masse* “burst” export events and MTOC-targeting phenotypes could be transferred to heterologous mRNAs using each distinct export element or tethered transport module, and were also observed for full-length HIV-1 and M-PMV gRNAs, respectively. Thus, mRNA export elements not only govern gRNA nuclear export but also pre-program gRNAs for distinct trafficking behaviors in the cytoplasm.

## Results

### 3-color visual system for studying HIV-1 gRNA nuclear export and virus particle assembly

**Fig 1:**
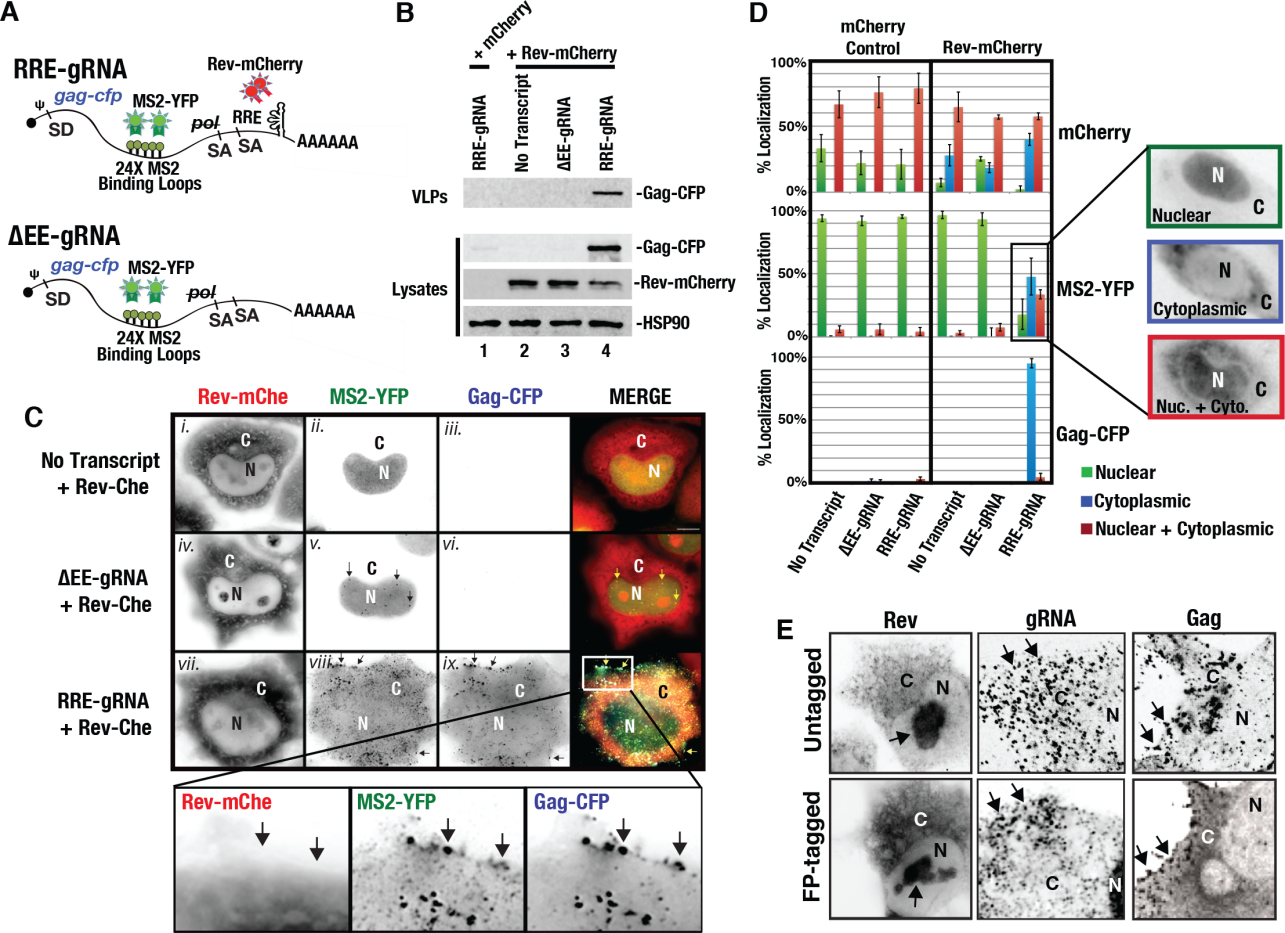
3-color system for studying HIV-1 gRNA trafficking. **(A)** Depiction of surrogate gRNA transcripts bearing either the RRE (RRE-gRNA) or lacking an export element (ΔEE-gRNA). Transcripts retained the native viral leader sequence including the packaging signal and include the major splice donor (SD) and two splice acceptors (SAs). **(B)** Virion-like particle (VLP) assembly assay. The indicated transcripts were co-expressed in Hela.MS2-YFP cells with either mCherry alone as a control (lane 1) or Rev-mCherry (lanes 2–4). Cells and supernatants were harvested at 48 h post-transfection. Cell lysates were processed for SDS-PAGE and immunoblot detection of p24^Gag^, mCherry, and HSP90 (loading control). VLPs were sedimented from supernatants by centrifugation through a sucrose cushion prior to SDS-PAGE and immunoblot detection of p24^Gag^. **(C)** Representative images of HeLa.MS2-YFP cells for an experiment as for (B) showing Rev-mCherry expressed either alone (*i*.*-iii*), with ΔEE-gRNA (*iv*.*-vi*.) or RRE-gRNA transcripts (*vii*.*-ix*.). Cells were fixed at 24 h post-transfection and imaged using deconvolution fluorescence microscopy. Arrows in panel *v*. highlight nuclear punctae corresponding to nucleus-restricted ΔEE-gRNA transcripts. Arrows in panel *viii*. highlight instances of marked co-localization between MS2-YFP and Gag-CFP signals at punctae at the plasma membrane consistent with assembling virus particles. Size bar represents 10 μm. (**D)** Quantification of subcellular distribution phenotypes. HeLa.MS2-YFP cells were transfected as for B and C to express the indicated transcripts in the presence of either a mCherry control or Rev-mCherry. Cells were fixed at 36 h post-transfection and scored for instances wherein the fluorescent signal was predominantly nuclear (green bars), cytoplasmic (blue bars) or distributed equally between the nucleus and cytoplasm (red bars). Error bars represent the standard deviation of the mean for 3 independent transfections (*n* ≈ 300 cells for each condition). Panels on the right show representative phenotypes for the MS2-YFP (gRNA) channel. (**E)** Control experiment demonstrating similar subcellular distributions for Rev, gRNA, and Gag detected with or without fluorescent protein tags. Untagged Rev and Gag in the top panels were detected by indirect immunofluorescence using mouse anti-Rev or anti-Gag antisera, respectively, followed by secondary anti-mouse antibodies conjugated to AlexaFluor594. Untagged gRNAs were detected using digoxigenin-labeled RNA probes complementary to sequences within the *gag* reading frame as described in Materials and Methods.

To study the effects of mRNA nuclear export elements on HIV-1 gRNA trafficking, translation, and virus particle assembly in single cells, we engineered visible virus particles generated from surrogate HIV-1 gRNAs encoding Gag fused in frame to CFP (Gag-CFP) and bearing 24 copies of the RNA MS2-binding loop (24xMBL) (Fig 1A). The MBL binds the MS2 bacteriophage coat protein with high affinity so that gRNAs are detected using co-expressed, nuclear-targeted MS2-YFP fusion proteins [68,69]. Our gRNAs carried the native 5’ untranslated region (UTR) including dimerization and packaging signals, the major splice donor, intact *gag-cfp* and *pol* coding regions, and two splice acceptors for *vif* and *vpr*, respectively (Fig 1A). Transcripts were truncated in the 3’UTR upstream of the *rev* and RRE coding regions allowing us to study gRNA trafficking with or without an RRE and/or Rev expression, as depicted (Fig 1A). Because the *gag* and *pol* genes are located within the major intron, Gag-CFP expression requires nuclear export of intron-containing, unspliced transcripts similar to those generated by full-length proviruses. Similarly, the 24xMBL cassette was positioned between the *gag-cfp* and *pol* coding regions so that only full-length, unspliced transcripts would be bound and labeled by MS2-YFP proteins. As expected, plasmids encoding RRE-containing gRNAs (RRE-gRNAs) or gRNAs lacking an export element (ΔEE-gRNAs) did not express Gag-CFP in HeLa cells modified to express nucleus-localized MS2-YFP constitutively (HeLa.MS2-YFP cells) (Fig 1B–1D). However, co-expression of RRE-gRNAs but not ΔEE-gRNAs with Rev-mCherry activated robust Gag-CFP synthesis and the assembly and release of virus-like particles (VLPs) (Fig 1B, compare Gag-CFP in lane 4 to lanes 1 and 3).

Using fluorescence microscopy, we detected discrete MS2-YFP punctae in the nucleus of cells expressing ΔEE-gRNAs or RRE-gRNAs in the absence of Rev (2.8-fold +/- 1.0 enrichment in mean fluorescence intensity relative to the surrounding nucleoplasm, *n* = 100) but not in the cytoplasm, consistent with visualization of gRNA transcripts incapable of leaving the nucleus in the absence of a competent nuclear export signal (*e*.*g*., Fig 1C, panel *v*.). By contrast, co-expression of RRE-gRNAs with Rev-mCherry dramatically shifted the MS2-YFP signal from the nucleus to the cytoplasm in most cells (74.3% +/- 4.72%, n = 297) correlating with robust Gag-CFP levels in the cytoplasm and, ultimately, the formation of bright punctae at the cell surface indicative of virus particle assembly (Fig 1C, panels *viii*. and *ix*., and Fig 1D). Notably, MS2-YFP-tagged gRNAs co-localized with Gag-CFP^+^ punctae at the plasma membrane, confirming that MS2-tagged gRNAs can successfully traffic with Gag-CFP to virus particle assembly sites (Fig 1C, panels *viii*. and *ix*., see boxed regions of interest), as previous demonstrated by others [70,71]. Moreover, we also evaluated the subcellular distribution of our Rev-mCherry, MS2-YFP, and Gag-CFP relative to untagged versions of each factor detected using immunofluorescence for Rev or Gag, or fluorescence in situ hybridization (FISH) using RNA probes that specifically bind full-length, *gag-pol* encoding gRNAs, and found them to be similar (Fig 1E). Taken together, these data and control experiments describe and validate a modular visual system for studying the effects of the RRE, Rev and CRM1 on HIV-1 gRNA trafficking, Gag expression, and virus particle assembly in single living cells.

### The HIV-1 Rev/RRE transport module regulates “burst” gRNA nuclear export dynamics

**Fig 2:**
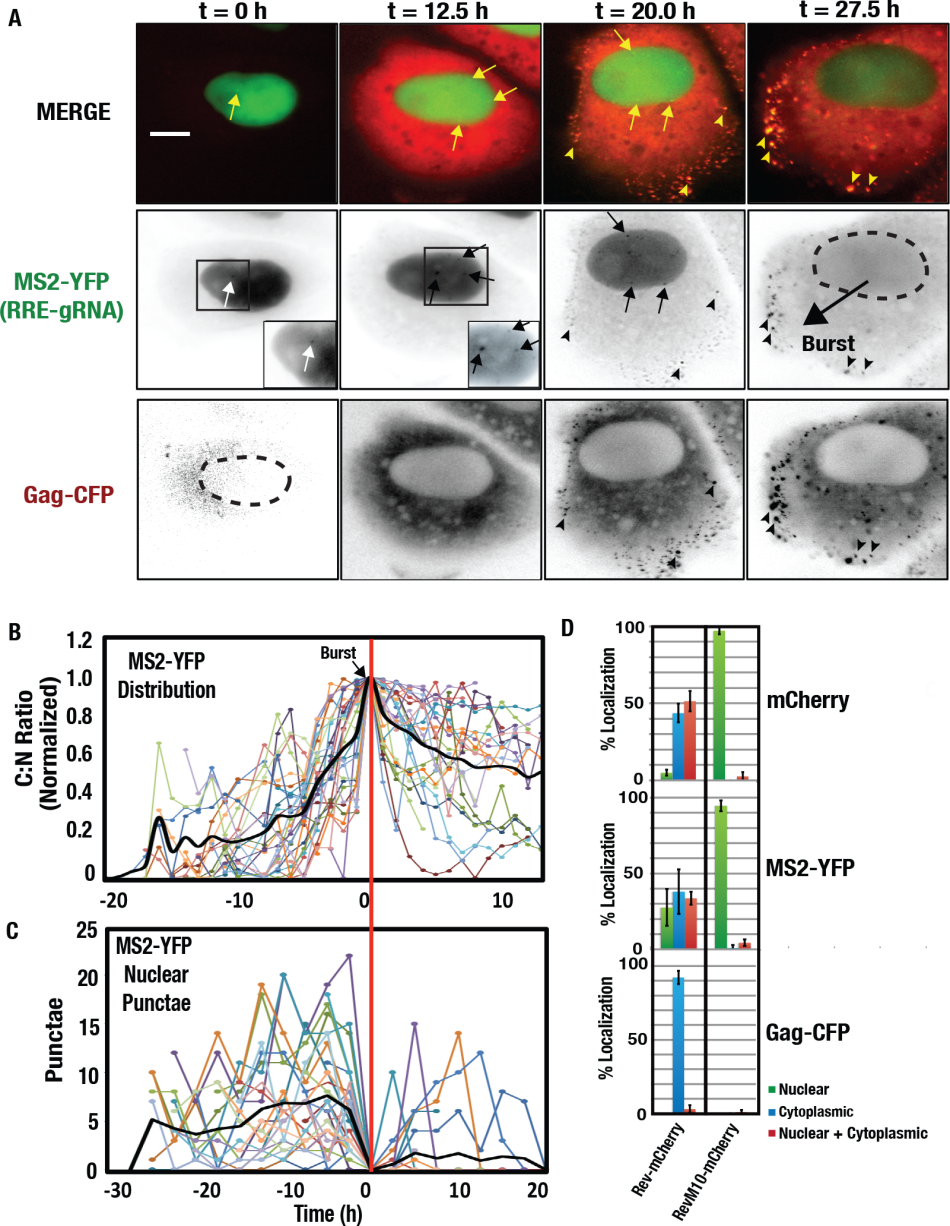
Rev- and CRM1-dependent “burst” HIV-1 gRNA nuclear export. **(A)** Select images from time lapse imaging showing MS2-YFP-tagged RRE-gRNAs and Gag-CFP subcellular distribution over 27.5 hours. “MERGE” shows Gag-CFP (pseudocolored red) and MS2-YFP tagged RRE-gRNA (green) with yellow arrowheads highlighting co-localization (yellow) of Gag and gRNA at the plasma membrane at later time points (20 and 27.5 hrs). The dotted line at the 27.5 hour panel for the MS2-YFP (RRE-gRNA) indicates the intact nuclear membrane after MS2-YFP has been evacuated from the nucleus. Size bar represents 20 um. **(B)** Burst export events. MS2-YFP tagged gRNA signals were measured in the cytoplasm and nucleus for 30 cells, normalized for peak cytoplasmic to nuclear (C:N) ratios of mean fluorescence intensity, and then aligned to this peak as t = 0. **(C)** Number of RRE-gRNA transcripts detected in the nuclear compartment of Hela.MS2-YFP cells before and after burst export for the cells measured for (B). **(D)** Quantification of subcellular distribution phenotypes as described for Fig 1E for RRE-gRNA transcripts expressed either with wild-type Rev-mCherry or a mutant Rev fusion protein (RevM10-mCherry) that cannot bind to CRM1. Error bars represent the standard deviation of the mean for 3 independent transfections (*n* ≈ 300 cells for each condition).

**Fig 3:**
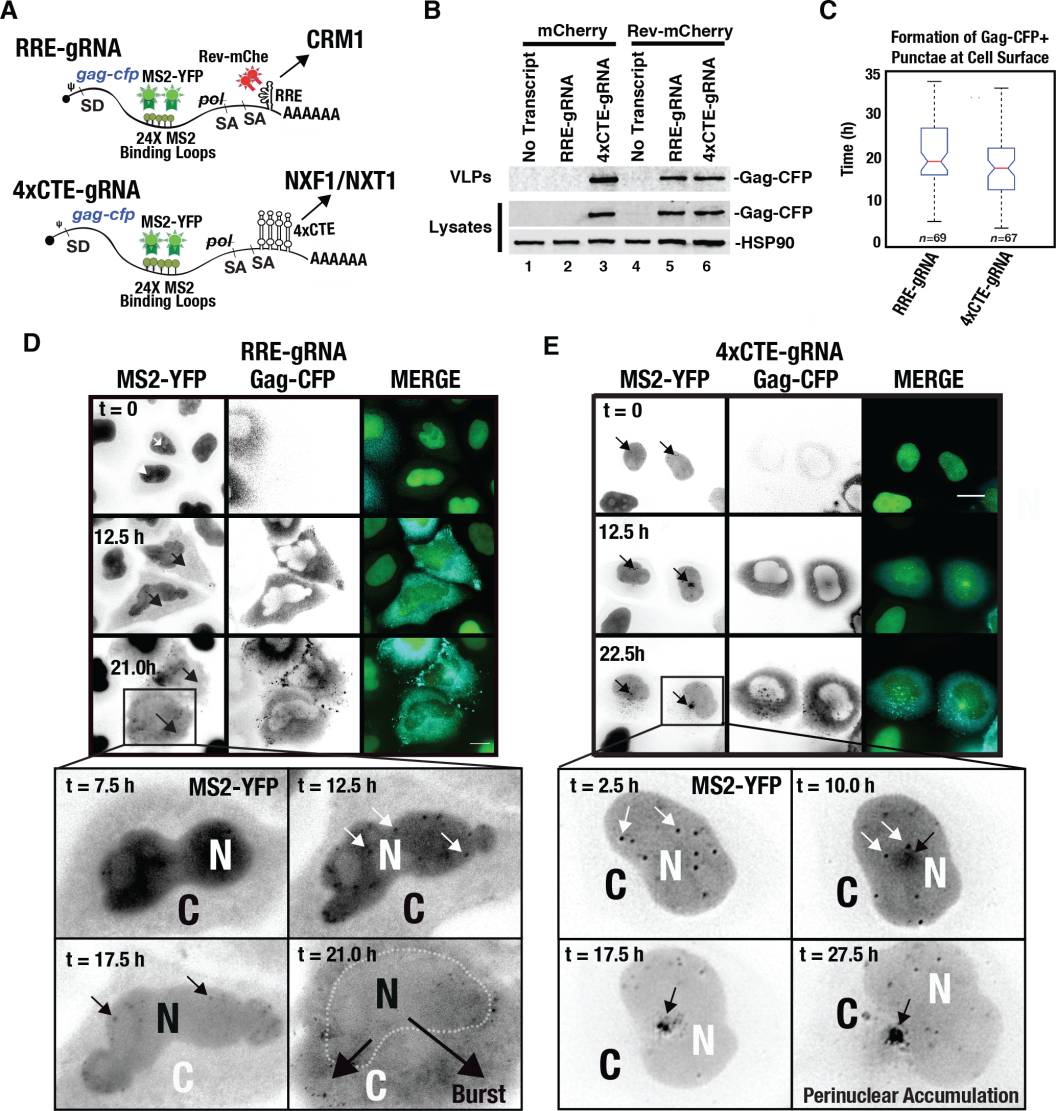
Differences to CRM1- and NXF1-dependent HIV-1 gRNA trafficking in the cytoplasm. **(A)** Depiction of RRE (Rev/CRM1) or 4xCTE (NXF1/NXT1)-dependent surrogate gRNA transcripts. **(B)** VLP assembly assay as described for Fig 1B showing Gag-CFP expression and virus particle assembly for either RRE- or 4xCTE-gRNA transcripts expressed in the presence or absence of Rev-mCherry. **(C)** Whisker plot of VLP production times acquired from live cell movies encompassing a >35 hour acquisition time for Gag-CFP derived from either RRE- or 4xCTE-gRNA transcripts. **(D)** Select images from time lapse imaging over >24 hours showing MS2-YFP-tagged RRE-gRNAs moving from the nucleus to the cytoplasm over time in conjunction with the onset of Gag-CFP expression and virus particle production. White arrows indicate individual transcripts in the nucleus. Black arrows represent the transition of the MS2-YFP signal from the nucleus to the cytoplasm in a burst-like fashion. The dotted line indicates the nuclear/cytoplasmic boundary post-burst. Figure corresponds to S1 Movie. **(E)** Unlike RRE-gRNAs, MS2-YFP-tagged 4xCTE-gRNAs accumulate at a perinuclear location coincident with the onset of Gag-CFP expression (12.5 h). White arrows indicate individual transcripts in the nucleus and black arrows highlight 4xCTE-gRNA perinuclear accumulation over time. Figure corresponds to S2 Movie. Size bars represent 10 μm.

Recent studies demonstrate that HIV-1 gRNA mobility in the cytoplasm is predominantly diffusive in nature [71–74], in contrast to earlier studies suggesting contributions from cytoskeletal elements and/or endosomal membranes to gRNA trafficking [75–77]. Collectively, these prior studies focused on fixed cells or visualized events in living cells over short time intervals (typically < 1 h). Thus, our goal was to monitor the entire productive phase of infection so that we carried out up to 30 hours of continuous multicolor single cell time lapse imaging (Figs 2 and 3 and S1 Fig). These experiments revealed that RRE-gRNAs formed MS2-YFP^+^ punctae within the nucleus as early as six hours post-transfection, followed by a Rev-triggered increase to cytoplasmic gRNA abundance and corresponding increases to Gag-CFP synthesis over time (Fig 2A, S1 Fig and S1 Movie). The gRNA transitions to the cytoplasm occurred quickly after the first detection of Rev-mCherry in the cell, consistent with RRE-dependent RNA nuclear export in response to low intracellular levels of Rev-mCherry (S1 Fig) [11]. Both gRNAs and Gag filled the volume of the cytosol in a non-localized fashion prior to aggregating together at later time points in the cell periphery, forming bright punctae consistent with the assembly of virus particles (Fig 2A). Because gRNAs and Gag flood the cytoplasm in a non-localized fashion prior to assembly, these observations are consistent with a diffusion-based model for gRNA/Gag trafficking in the cytosol.

We found it notable that, in greater than 30% of Rev-expressing cells, we observed a nearly complete relocalization of the MS2-YFP signal from the nucleus to the cytoplasm over time (Figs 1D, 2A and 2B and S1 Movie). Because the MS2-YFP signal is a proxy for gRNA localization, such observations would be best explained by RRE-gRNAs saturating all MS2-YFP proteins in the nucleus prior to rapidly transferring them to the cytoplasm. In many cases this transition was gradual, with a shift in minimum to maximum cytoplasmic to nuclear (C:N) ratio of MS2-YFP mean fluorescence intensity (MFI) occurring over ~9.0 hours (+/- 3.4 h, *n* = 30) (Fig 2B). However, many cells exhibited more striking, “burst-like” export dynamics wherein the MS2-YFP signal was evacuated from the nucleus *en masse* much more rapidly, in less than an hour (Fig 2A, right panels, 2B and S1 Movie). In these cells the number of nuclear MS2-YFP punctae increased just prior to nuclear evacuation (n = 30) (Fig 2C), prompting us to test if the “burst” reflected a threshold-triggered gRNA nuclear export event regulated by CRM1. Indeed, MS2-YFP was never observed in the cytoplasm when RRE-gRNAs were transfected with a mutant form of Rev (RevM10-mCherry) that is capable of binding to the RRE but not to CRM1 due to a disrupted NES peptide [14,78] (Fig 2D). Thus, Rev-CRM1 interactions play a crucial role in regulating HIV-1 burst export dynamics.

### Replacing the RRE with multiple copies of the CTE re-programs HIV-1 gRNAs to accumulate at the microtubule organizing center

Having defined a unique, CRM1-linked nucleocytoplasmic trafficking behavior for HIV-1 surrogate gRNAs, we next sought to compare the CRM1 pathway to the more conventional mRNA nuclear export pathway regulated by NXF1/NXT1. To this end, we replaced the RRE in our HIV-1 gRNA transcripts with one or multiple copies of the CTE derived from M-PMV (4xCTE-gRNA depicted in Fig 3A). The M-PMV CTE is a structured RNA element that binds to a heterodimer of NXF1/NXT1 [29,30,54–56,79]. Consistent with earlier studies [29,30], replacing the RRE with a single CTE (1xCTE-gRNA) yielded only low levels of HIV-1 gRNA nuclear export and Gag-CFP synthesis (S2 Fig panel A), reflecting the activity of strong export inhibitory sequences (so-called “INS” elements) found within the *gag* reading frame [80]. However, we found that in HeLa.MS2-YFP cells four copies of the CTE (4xCTE-gRNA) activated Rev-independent Gag-CFP expression to levels roughly equivalent to RRE-gRNAs expressed in the presence of Rev-mCherry **(** Fig 3B, compare lanes 5 and 6), thus providing us with a suitable comparator.

Time-lapse video microscopy confirmed similar rates of Gag-CFP punctae formation for either pathway in single cells, appearing at the plasma membrane as early as 5 hours post-transfection for either pathway (16.7 h +/- 8.06 h for 4xCTE and 20.3 h +/- 8.07 h for RRE+Rev; Fig 3C–3E, S1 Movie, and S2 Movie). Interestingly, single cell measurements of Gag-CFP cytoplasmic abundance over 24 hours demonstrated that RRE-derived Gag-CFP increased quickly at the onset of Rev-mCherry expression and then slowed between 10 and 15 hours post-transfection, unlike 4xCTE-derived Gag-CFP or a CFP alone control that rose in a more linear fashion over the time course (S2 Fig panel B). Despite these similar overall Gag-CFP expression profiles, we observed remarkable differences to the trafficking behaviors of RRE-gRNAs compared to 4xCTE-gRNAs. Unlike RRE-gRNAs co-expressed with Rev-mCherry that exhibited burst export dynamics and dispersed throughout the cytoplasm (Figs 2A and 3D and S1 Movie), 4xCTE-gRNAs did not “burst” but instead clustered gradually over time to regions at or near the nuclear membrane, ultimately forming a dense cluster in the cytoplasm (Fig 3E and S2 Movie). HIV-1 Gag-CFP was also observed to accumulate at the dense cluster over time, suggesting localized translation or retention of Gag-CFP at this site (Fig 3E and S2 Movie and S2 Fig, panel C).

**Fig 4:**
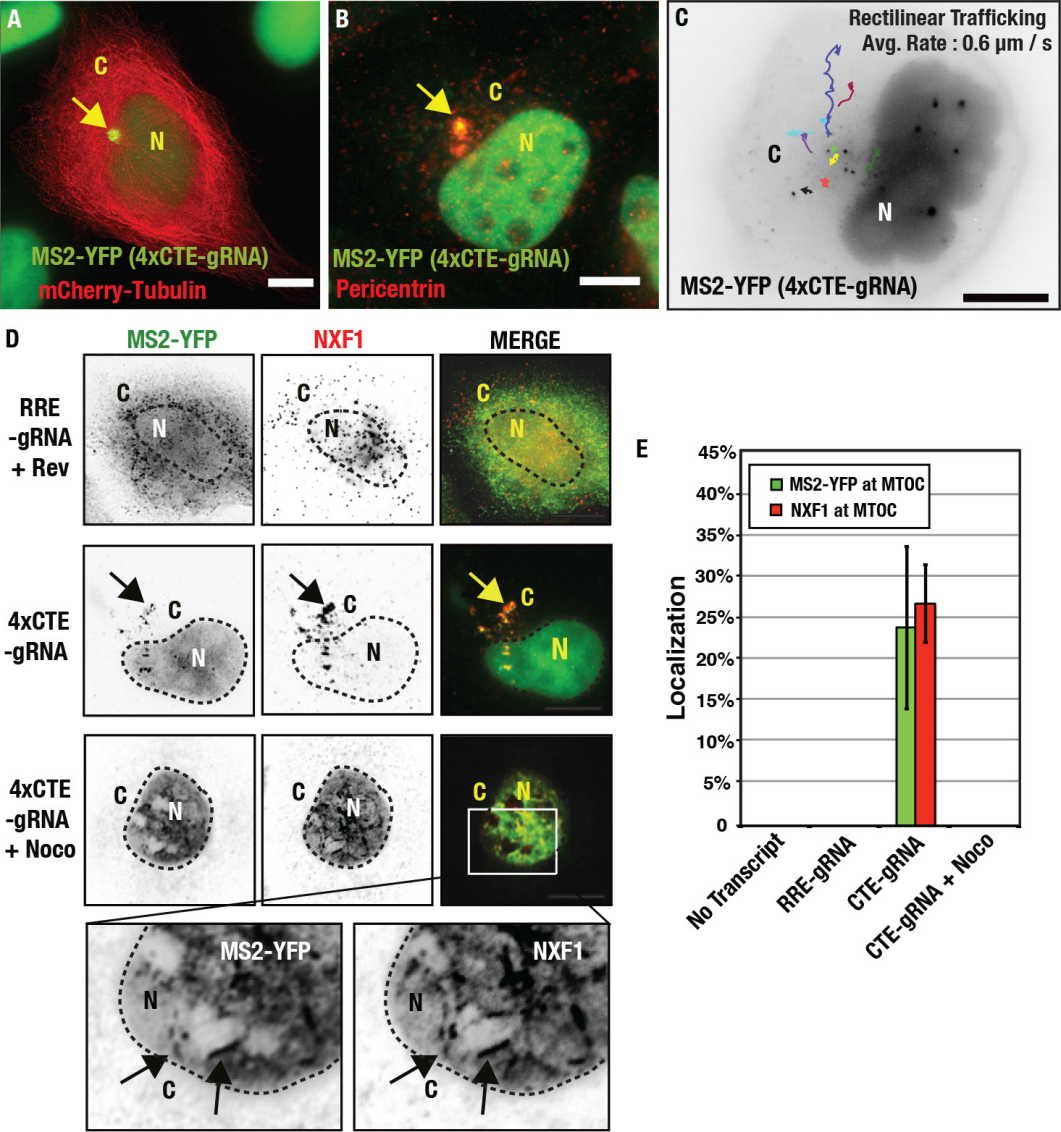
CTE-gRNAs accumulate at the microtubule organizing center. **(A, B).** 4xCTE-gRNAs in HeLa.MS2-YFP cells cluster at the MTOC as demonstrated by co-expressing mCherry-Tubulin **(A)** or staining for the centrosomal marker Pericentrin **(B)**. Arrows highlight the marked enrichment of CTE-gRNAs to this region. **(C)** Single particle tracking over 10 minutes for 4xCTE-gRNAs undergoing bidirectional and rectilinear trafficking at or near the MTOC. Arrows represent individual gRNA paths over the time of imaging. Figure corresponds to S4 Movie. **(D)** NXF1 traffics with 4xCTE-gRNAs to the MTOC. Cells expressing RRE- or 4xCTE-gRNAs were incubated with or without 3 μM nocodazole (as indicated) for 36 h prior to fixation and detection of NXF1 using indirect immunofluorescence. Black arrows indicate regions of co-localization between NXF1 and 4xCTE-gRNAs **(E)** Quantification of MS2-YFP and NXF1 localization at the MTOC for the indicated conditions. For these experiments the MTOC was defined using Pericentrin immunostaining. Error bars represent the standard deviation of the mean for three (MS2-YFP, *n* = 50) or two (NXF1, *n* = 50) independent experiments. All size bars correspond to 10 μm.

Based on morphology, we hypothesized that 4xCTE-gRNA transcripts were accumulating at the microtubule-organizing center (MTOC), a perinuclear organelle that nucleates microtubule (MT) polymerization and anchors the MT network [81–83]. We confirmed that 4xCTE-gRNAs were clustered to the MTOC by co-expressing mCherry-tagged alpha-tubulin (Fig 4A and S3 Movie) or by detecting Pericentrin, a marker for the centrosome, a mass of regulatory signaling proteins that surrounds specialized MTs known as centrioles [84,85] (Fig 4B). Although 4xCTE-gRNAs frequently clustered in close proximity to the centrosome, this clustering was dynamic, with MS2-YFP signals exhibiting bi-directional transport in apparent association with MTs at or near the MTOC at rates consistent with MT-based motors (0.6 μm / s +/- 0.87) (Fig 4C, S3 Movie and S4 Movie). NXF1 also co-clustered with 4xCTE-gRNAs at the MTOC as detected using indirect immunofluorescence (Fig 4D, middle panels, and Fig 4E). Treatment of cells with nocodazole, a drug that depolymerizes MTs, completely abolished 4xCTE-gRNAs clustering to the MTOC (Fig 4E) and arrested both transcripts and NXF1 at the nuclear membrane (Fig 4D, bottom panels, Fig 4E). Thus, 4xCTE-gRNAs and NXF1 apparently exploit MTs to co-traffic from the nuclear membrane to the centrosome.

**Fig 5:**
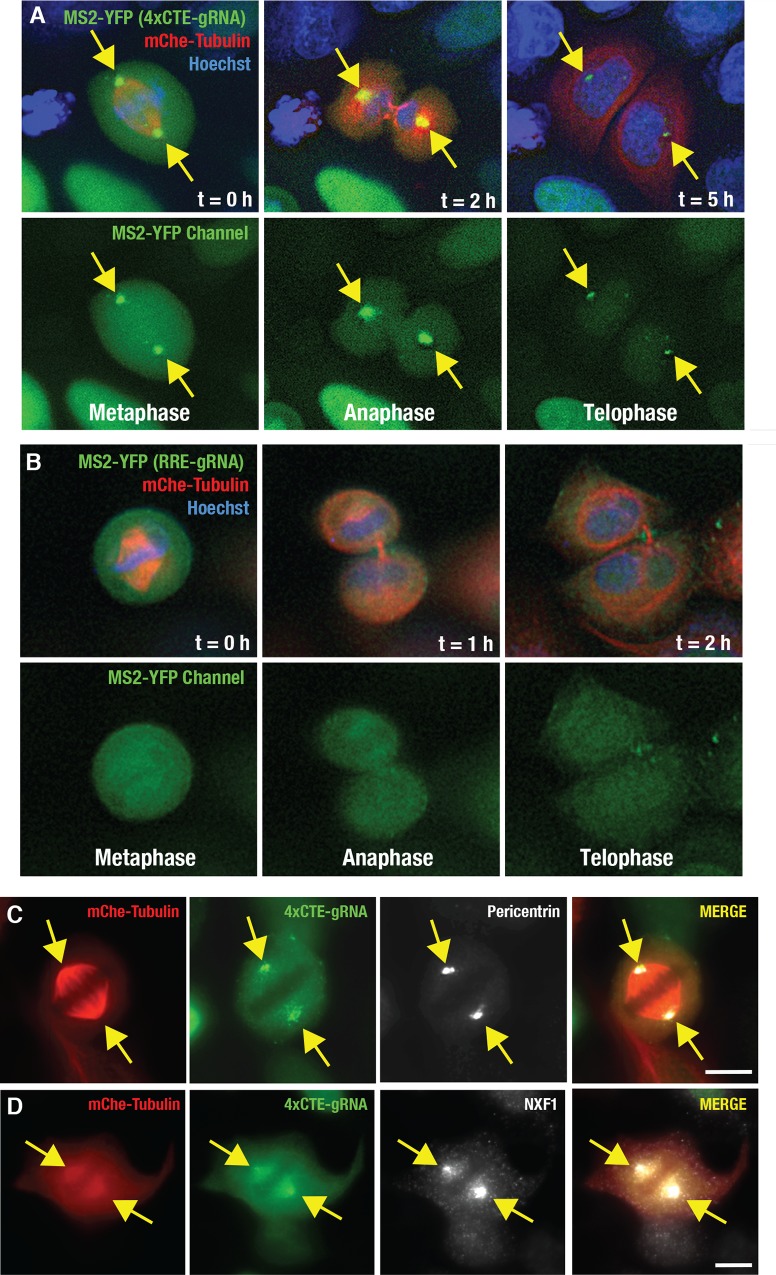
CTE-gRNAs accumulate at centrosomes during mitosis and are subsequently partitioned to daughter cells. **(A)** 4xCTE-gRNAs are partitioned to daughter cells via centrosomes during cell division. A representative HeLa.MS2-YFP cell expressing 4xCTE-gRNAs and tracked through metaphase, anaphase, and telophase. Yellow arrows indicate the enriched MS2-YFP signal (green) at the spindle poles (mCherry-tubulin in red) during metaphase (Hoechst DNA stain in blue). Figure corresponds to S5 Movie. **(B)** Unlike 4xCTE-gRNAs, RRE-gRNAs do not accumulate at the mitotic spindle. Imaging as for (A) but for cells co-expressing RRE-gRNAs and Rev. **(C,D)** 4xCTE-gRNAs (MS2-YFP; green) co-localize with the centrosome marker Pericentrin (C, white) and also NXF1 (D, white) at the poles of the spindle (shown using mCherry-tubulin, red). Pericentrin and NXF1 were detected by indirect immunofluorescence. For all panels, yellow arrows highlight 4xCTE-gRNA enrichment at the centrosome. Size bars represent 10 μm.

Remarkably, in actively dividing cells, we also observed 4xCTE-gRNAs accumulating to high levels at the duplicated centrosomes that form the poles of the mitotic spindle (Fig 5A, 5C and 5D and S5 Movie). At the onset of M phase, centrioles double, separate, and direct the formation of the mitotic spindle apparatus that mediates nuclear membrane breakdown and the separation of sister chromatids [86]. 4xCTE-gRNAs remained associated with centrosomes during sister chromatid partitioning (anaphase), reformation of the nuclear membrane (telophase), and completion of cytokinesis, and then ultimately dissipated into the cytosol of each daughter cell (Fig 5A, panels moving left to the right, and S5 Movie). By contrast, RRE-gRNAs were never observed in association with the spindle, even despite CRM1’s known role as an effector of mitosis that associates strongly with spindle MTs (Fig 5B) [87]. In sum, the 4xCTE but not the RRE traffics gRNAs to centrosomes during interphase and also in conjunction with nuclear membrane breakdown and mitotic spindle formation during cell division.

### Burst and MTOC trafficking behaviors are intrinsic to RRE/Rev and CTE/NXF1/NXT1 export modules, respectively

**Fig 6:**
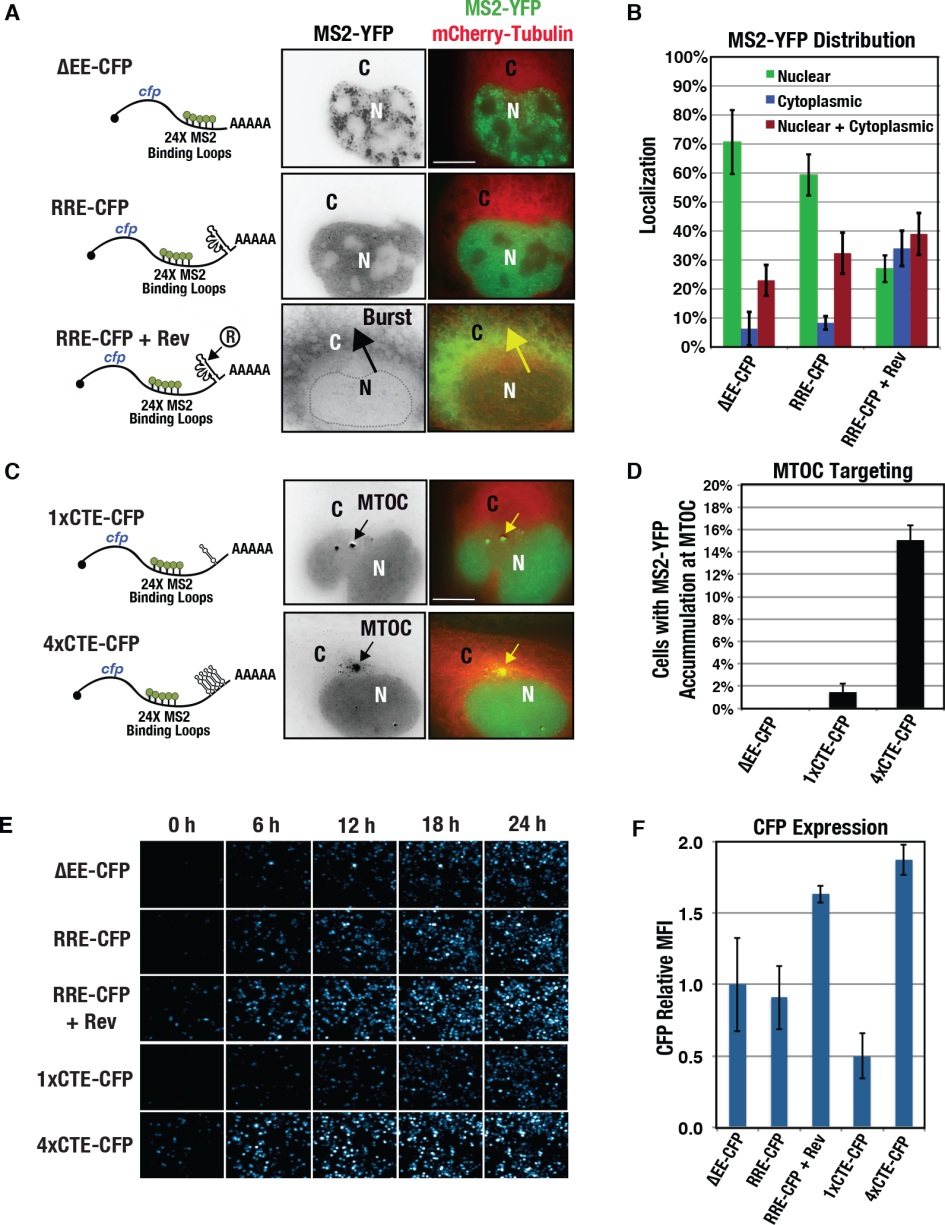
The RRE and CTE are both necessary and sufficient to trigger burst and MTOC-trafficking phenotypes, respectively. **(A)** Depiction of heterologous, 24xMBL bearing ΔEE- and RRE-CFP transcripts and representative visual phenotypes in cells also expressing mCherry-Tubulin (red) as an MTOC marker. The MS2-YFP signal (green) was high within the nucleus for cells expressing ΔEE-CFP transcripts and RRE-CFP transcripts expressed in the absence of Rev. However, Rev triggered the relocalization of RRE-CFP transcripts to the cytoplasm. **(B)** MS2-YFP subcellular distribution for the indicated conditions as described for Fig 1E. **(C)** Depiction of heterologous, 24xMBL-bearing 1xCTE- and 4xCTE-CFP transcripts and representative visual phenotypes as for (A). **(D)** MTOC-targeting analysis as for Fig 4E for the indicated conditions. (**E)** Selected frames from time-lapse imaging demonstrating CFP expression over time for all indicated model transcripts. Images are low magnification and show >100 cells per frame. **(F)** Quantification of CFP mean fluorescence intensity (MFI) for all conditions in (E) recorded at 24 hours. All size bars represent 10 μm.

We next asked if the RRE or CTE were sufficient to transfer burst export or MTOC-trafficking behaviors to non-viral, heterologous transcripts. To this end, we engineered each export element (RRE, 1xCTE, and 4xCTE) into minimal transcripts encoding only CFP and bearing the 24xMBL cassette within the 3’UTR (Fig 6A). These transcripts lacked introns and were thus export-competent and expressed CFP with or without the addition of viral elements (see Fig 6E and 6F). Indeed, control transcripts lacking an export element (ΔEE-CFP) triggered the formation of MS2-YFP punctae in the nucleus and delivered low levels of MS2-YFP signal to the cytoplasm (Figs 6A, top panels, and 6B). Similar results were obtained for otherwise identical transcripts carrying the HIV-1 RRE (RRE-CFP) but expressed in the absence of Rev (Fig 6A, middle panels and 6B). However, the provision of Rev to RRE-CFP transcripts resulted in burst export in more than 30% of cells (Fig 6A, lower panels, and 6B), a result identical to that observed for RRE-gRNA intron-containing transcripts (Figs 1–3). We also found that a single CTE was sufficient to target CFP transcripts (1xCTE-CFP) to the MTOC, albeit to a lesser extent than the 4xCTE cassette (Fig 6C and 6D). Thus, multiple CTEs exert additive effects in targeting export-competent mRNAs to the MTOC. Interestingly, the RRE/Rev and 4xCTE conditions both enhanced CFP expression almost two-fold relative to the ΔEE-CFP control (Fig 6E and 6F), suggesting enhanced nuclear export kinetics or other effects on mRNA cytoplasmic utilization attributable to these export elements. Taken together, these experiments revealed that the Rev/RRE and CTE/NXF1 export systems are both necessary and sufficient to activate burst and MTOC-directed mRNA trafficking behaviors.

**Fig 7:**
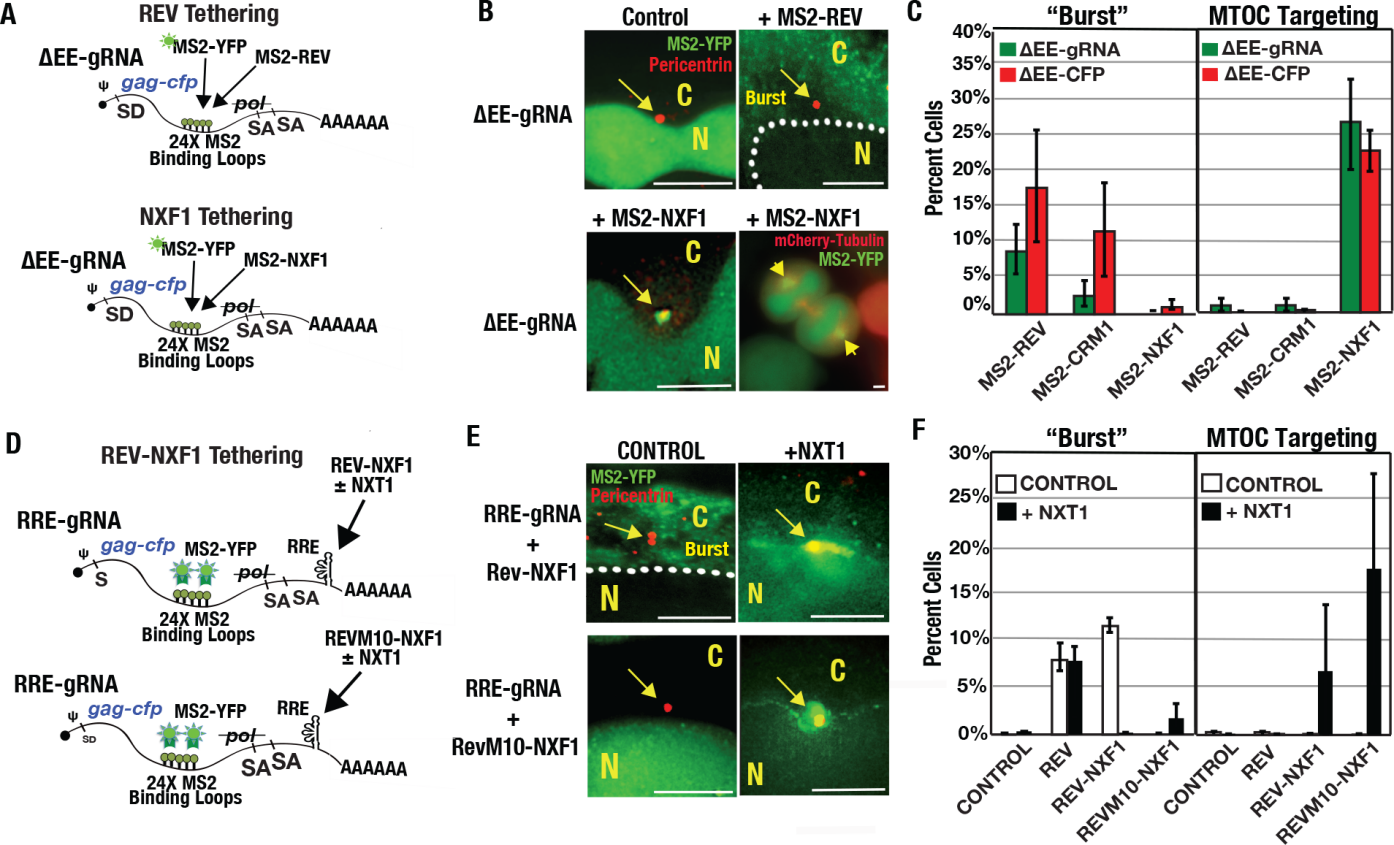
The Rev/CRM1 or NXF1/NXT1 export modules are sufficient to trigger RNA burst export or MTOC targeting, respectively, and independently of an export element. **(A)** Depiction of 24xMBL-bearing ΔEE HIV-1 gRNAs expressed with or without MS2-tagged fusion proteins in HeLa.MS2-YFP cells. **(B)** MS2-Rev expression led to ΔEE-gRNA evacuation from the nucleus (MS2-YFP signal in green), while MS2-NXF1 expression directed transcripts to the MTOC (yellow arrows, Pericentrin stain in red). MS2-YFP signal is also shown detected for ΔEE-gRNAs expressed with MS2-NXF1 at centrosomes (yellow arrows) in mitotic cells expressing mCherry-Tubulin (red). **(C)** Quantification of burst export and MTOC-targeting phenotypes for each condition as measured for Figs 1D and 4E. **(D)** Depiction of 24xMBL-bearing ΔEE HIV-1 gRNAs expressed with or without Rev/RevM10-NXF1 fusion proteins. **(E)** As for (B), burst or MTOC-targeting for RRE-gRNAs expressed with Rev-NXF1 or RevM10-NXF1 fusion proteins, with or without NXT1 as indicated. **(F)** Quantification as for (C) for the experiments depicted in (D) and (E). All size bars represent 5 μm.

Because the multimeric CTE was more active than a single CTE, we hypothesized that mRNA clustering to the centrosome reflected the extent of interactions occurring between single mRNA transcripts and one or more NXF1/NXT1 heterodimers (Fig 7). As demonstrated for MS2-YFP, MS2 coat proteins effectively tether heterologous protein domains to 24xMBL-bearing transcripts [88,89]. Therefore, we tested the effects of tethering ΔEE-gRNA or ΔEE-*cfp* model transcripts to previously validated MS2-Rev, MS2-CRM1, and MS2-NXF1 fusion proteins [90,91] (Fig 7A). MS2 coat proteins form dimers so that up to 48 MS2-tagged proteins are capable of interacting with a single transcript in this experiment. Although less active than the native Rev/RRE cassette (see Fig 1), MS2-Rev expression led to burst export in 8.9% (±3.21%) or 17.9% (±7.9%) of cells expressing ΔEE-gRNA or ΔEE-cfp transcripts, respectively, evaluated at 24 h post-transfection (Fig 7B and 7C). MS2-CRM1 was also sufficient to trigger burst export, although it was more active for ΔEE-cfp transcripts than for ΔEE-gRNAs (Fig 7C). By contrast, MS2-NXF1 expression did not trigger bursts, and instead directed transcripts to the MTOC in greater than 20% of transfected cells for either transcript (Fig 7B and 7C). These results demonstrated that Rev/CRM1 or NXF1 tethering is sufficient to drive burst and cluster RNA transcripts to the MTOC, respectively.

We also tested the effects of directly tethering NXF1 to the RRE by expressing Rev-NXF1 or RevM10-NXF1 fusion proteins (Fig 7D). We predicted that Rev-NXF1 binding to the RRE would lead to competition between the CRM1 and NXF1/NXT1 pathways, while inactivating Rev’s NES (RevM10-NXF1) would abolish CRM1 recruitment but still allow for interactions with NXT1. Importantly, Guzik *et al*., previously showed that RevM10-NXF1 fusions are sufficient to activate RRE-dependent, CRM1-independent gene expression [92]. However, this activity was only observed when expressing supraphysiological levels of NXT1 [92]. Interestingly, in our experiment Rev-NXF1 expression yielded burst export events equivalent to a Rev control (11.5% of cells ±0.5%), while the addition of NXT1 completely abolished burst export and re-routed these transcripts to the MTOC in 6.8% of cells (±6.2%) (Fig 7E and 7F). Similarly, the RevM10-NXF1 fusion protein had no activity on its own but, when co-expressed with NXT1, targeted RRE-gRNAs to the MTOC with efficiency in 17.7% of cells (±10.1) (Fig 7E and 7F). These experiments further confirmed that burst export and MTOC targeting are intrinsic to each distinct export modules.

### Full-length HIV-1 and M-PMV gRNAs exhibit burst export and MTOC-trafficking phenotypes, respectively

**Fig 8:**
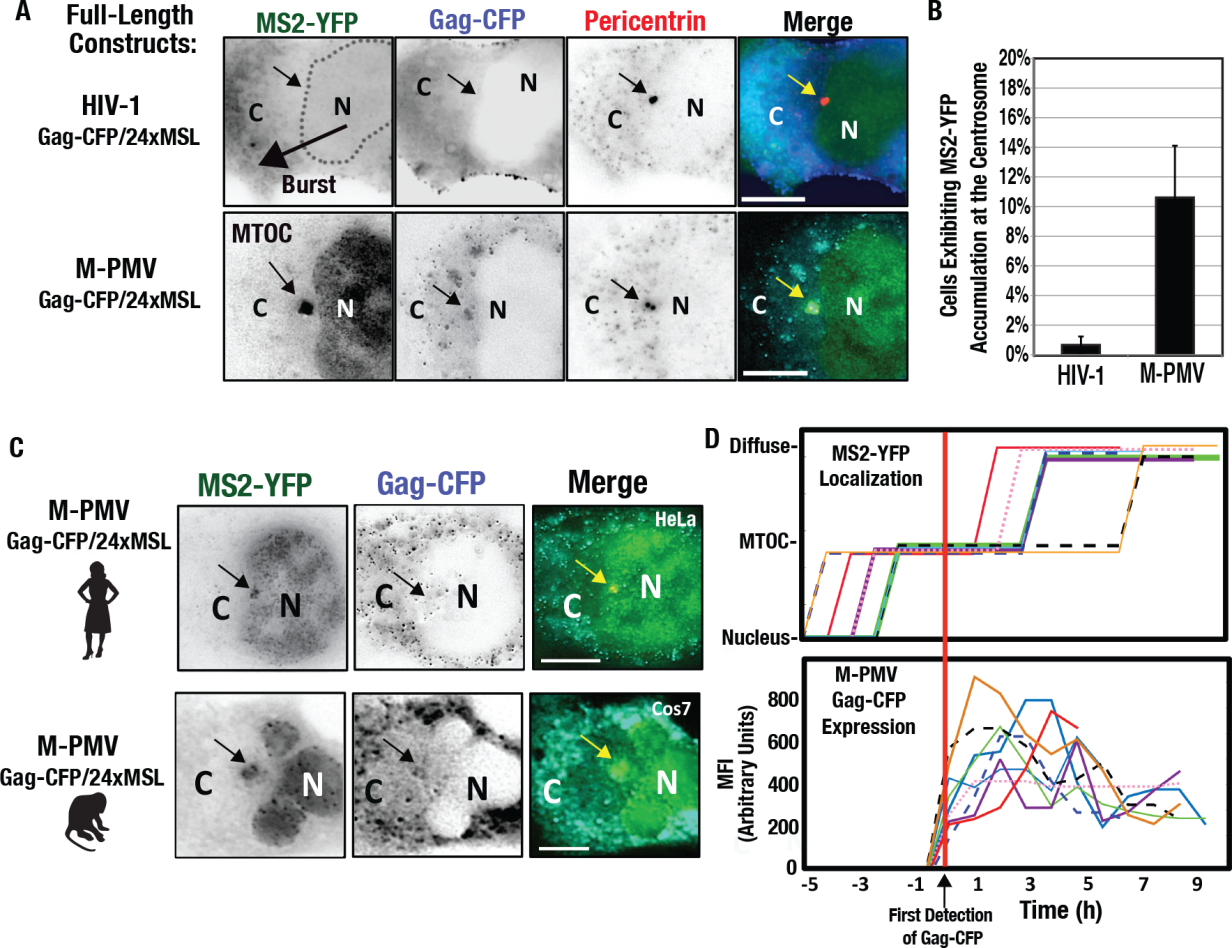
Full Length HIV-1 and M-PMV gRNA exhibit burst nuclear export and MTOC-targeting phenotypes, respectively. **(A)** Representative images of full-length HIV-1 and M-PMV reporter viruses modified to carry the 24xMBL cassette and express Gag-CFP (blue) in HeLa.MS2-YFP cells at 24 hours post-transfection. Dotted line indicates nuclear/cytoplasmic boundary after gRNA (green) was evacuated from the nucleus. Black and yellow arrows indicate the location of the centrosomes based on Pericentrin (red) immunofluorescence. **(B)** MTOC-targeting for HIV-1 and M-PMV gRNAs as measured for Fig 4E. **(C)** M-PMV gRNAs accumulated at the MTOC in both human HeLa (top panels) and African green monkey Cos7 cells (bottom panels). **(D)** M-PMV gRNA clustering at the MTOC precedes Gag-CFP synthesis. Gag-CFP synchronized rise in mean fluorescence intensity (MFI) over time (lower panel, *n* = 10 cells) relative to the defined subcellular distribution of MS2-YFP for the same cells (top panel). All size bars represent 10 μm.

Based on the observations above, it was important to determine if these distinct trafficking behaviors were recapitulated in full-length HIV-1 and M-PMV gRNAs. To address this question, we engineered full-length HIV-1 [93,94] and M-PMV [95,96] reporter viruses modified to similarly express Gag-CFP and also carry the 24xMBL cassette within the major intron (Fig 8). As anticipated, full-length Gag-CFP/24xMBL HIV-1 gRNAs expressed in HeLa.MS2-YFP cells evacuated the nucleus *en masse* to flood the cytoplasm in conjunction with increases to Gag-CFP synthesis and prior to virus particle assembly (Fig 8A, top panels, and S6 Movie).

By contrast, M-PMV gRNAs accumulated near the nuclear membrane, clustering to the MTOC in greater than 10% of transfected cells evaluated at 24 h post-transfection (Fig 8A and 8B and S7 Movie **).** We observed M-PMV gRNA MTOC-targeting in both human and non-human primate (Cos7) cell types (Fig 8C). Interestingly, in fixed cells we frequently observed M-PMV gRNAs at the MTOC both without co-clustering of its protein product, Gag-CFP (*e*.*g*., compare Fig 8A, lower panels to Fig 8C, upper panels). To explain this observation, we performed live imaging that revealed that M-PMV gRNA MTOC-targeting almost invariably preceded the onset of detectable Gag-CFP synthesis (Fig 8D). Thus, M-PMV gRNA trafficking to the MTOC is both intrinsic to native gRNAs and is apparently one of the earliest events of the viral productive phase.

## Discussion

Herein we describe a novel long-term (>24 h), modular, multicolor imaging strategy for visually dissecting the integrated stages of HIV-1 gRNA transcription, nuclear export, Gag translation, and virus particle assembly (Fig 1). We use this approach to compare mRNA trafficking behaviors for HIV-1 intron-bearing gRNA transcripts in either the CRM1 or NXF1/NXT1-dependent nuclear export pathways (Figs 1–7) and also compared full-length HIV-1 gRNAs to M-PMV gRNAs (Fig 8). We show that Rev-dependent mRNAs are frequently programmed to leave the nucleus *en masse*, flooding the cytoplasm in conjunction with Gag-CFP translation (Figs 2A, 3D, 6A and 8A, S1 Movie and S6 Movie). In contrast, CTE-bearing transcripts become linked to microtubules in the cytoplasm and traffic rapidly to the MTOC to cluster at centrosomes in both interphase and metaphase cells (Figs 3E, 4, 5A, 6C and 8A, S2 Movie, S3 Movie, S4 Movie, S5 Movie and S7 Movie).

**Fig 9:**
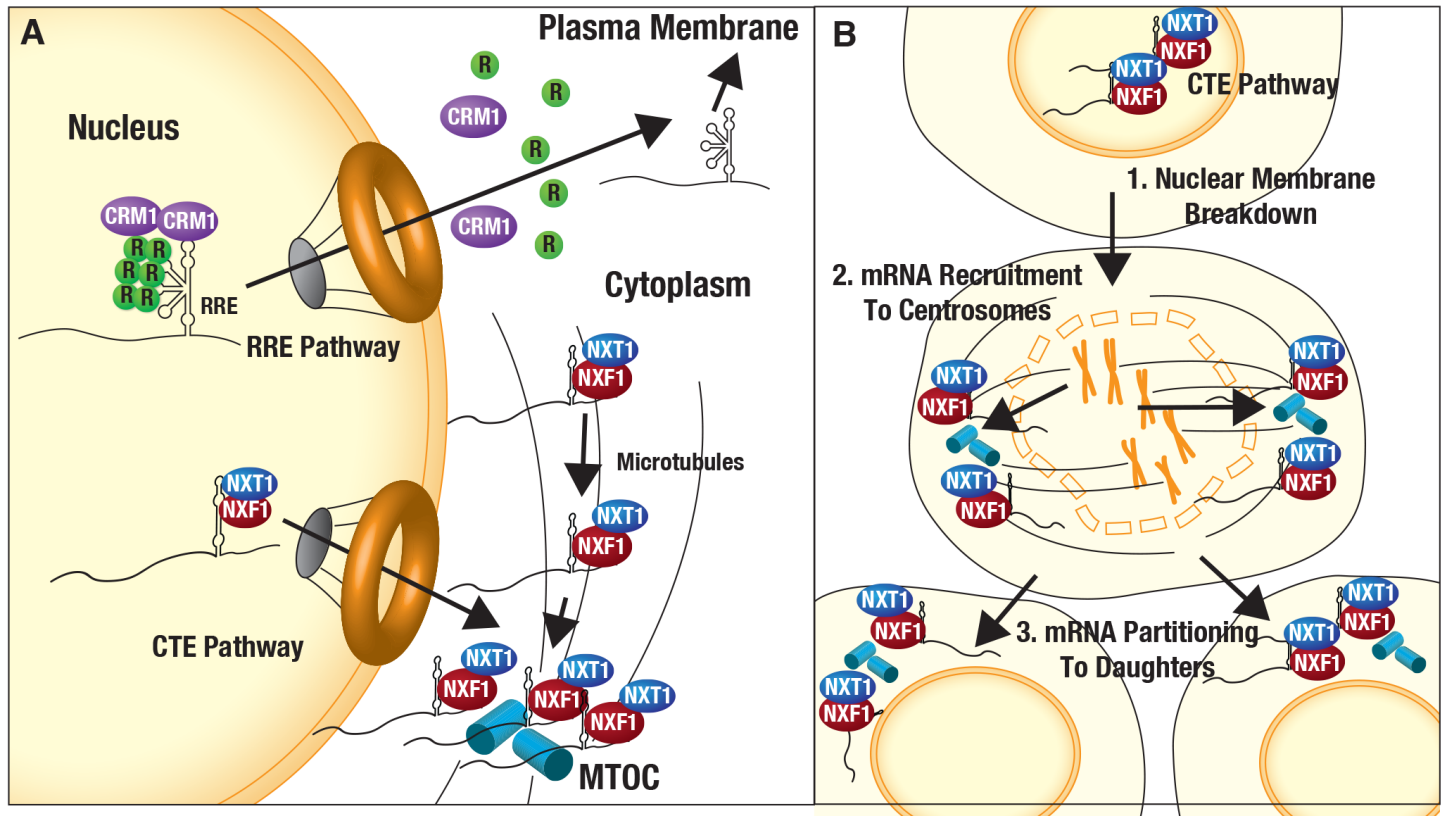
Working model for linkages between retroviral gRNA nuclear history and trafficking in the cytoplasm. **(A)** In interphase cells, CRM1 regulates punctuated transitions of RRE-gRNAs from the nucleus to flood the cytoplasm in conjunction with Gag expression and the onset of virus particle assembly at the plasma membrane. 4xCTE-gRNAs (and M-PMV gRNAs) also leave the nucleus through the nuclear pore complex but instead are linked to microtubules that direct their trafficking to the microtubule organizing center (MTOC) and centrosome. **(B)** 4xCTE-gRNAs are also targeted to centrosomes during cell division. At the onset of mitosis, the nuclear membrane breaks down (phase 1) and 4xCTE-gRNAs are rapidly directed to duplicated centrosomes that form the poles of the mitotic spindle during metaphase (phase 2). Subsequently, CTE-gRNAs bound to centrosomes are partitioned to daughter cells and then released into the cytoplasm for Gag synthesis (phase 3).

Two decades of research have clearly established that export elements regulate retroviral gRNA nuclear export and are essential to viral late gene expression [21,22]. Our data reveal, for the first time, that export elements also program unique gRNA trafficking behaviors in the cytoplasm. HIV-1 is a C-type retrovirus that assembles virus particles at the plasma membrane [28,97,98]. By contrast, M-PMV is a D-type retrovirus that assembles its capsids preferentially in the cytoplasm [99–101]. In Fig 9 we propose a working model for HIV-1 and M-PMV gRNA trafficking wherein HIV-1 preferentially exploits the Rev/RRE/CRM1 pathway in order to flood the cytoplasm with gRNAs during the late, productive stages of infection. Increases to gRNA cytoplasmic abundance coincide with increases to Gag synthesis (S1 Fig) and we speculate also encourage the formation of Gag/gRNA transport complexes that subsequently migrate to the plasma membrane (Fig 9A, top). By contrast, M-PMV gRNAs target the MTOC even prior to Gag synthesis (Fig 8F), perhaps priming the cell for viral capsid biogenesis in the perinuclear region (Fig 9A, bottom). Consistent with this notion, Sfakianos *et al*. previously suggested that M-PMV capsids are surrounded by an abundance of ribosomes at the MTOC, and sit in close proximity to recycling endosomes bearing viral glycoproteins [102]. Thus, an attractive model is that the MTOC compartmentalizes Gag/Gag-Pol synthesis prior to “loading” nascent capsids onto recycling transport vesicles bound for the cell surface. Consistent with this notion, M-PMV trafficking and virion production have been shown to exhibit sensitivity to pharmacological perturbations of the MT cytoskeleton [95,96] while HIV-1 assembly is reported to be largely unaffected [103].

Several other exogenous and endogenous retroviruses as well as additional viral pathogens including African swine fever virus, noroviruses, poxviruses, and herpesviruses are also thought to exploit the MTOC to coordinate aspects of their virion assembly pathways [104–112]. Because viruses frequently co-opt pre-existing cellular mechanisms, it is compelling to speculate that NXF1/NXT1 plays a general role in coordinating viral and cellular mRNAs to the centrosome. Our mRNA tethering experiments suggest that the capacity of NXF1/NXT1 to direct mRNAs to the MTOC is a function of the extent of NXF1/NXT1 binding (Fig 6D), the abundance of relevant co-factors (e.g., NXT1; Fig 7F), and can be transient (perhaps best illustrated during mitosis, see Fig 5A). However, it is important to note that not all retroviruses that encode CTEs assemble in the cytoplasm, for example MLV encodes a CTE but uses a C-type assembly pathway [31–33,113]. Conversely, human foamy virus gRNAs [105] and a subset of influenza A virus (-) strand genomic RNA segments [114,115] accumulate at the MTOC despite reported links to CRM1 [116–118]. Thus, although we demonstrate that NXF1/NXT1 is sufficient to target select mRNAs to the MTOC, additional *cis*- and *trans*-acting signals are likely to cooperate with these factors in ways that determine if, how, and when a particular mRNP is trafficked and translated. Additional comparative studies will be essential to further elucidate the unique and shared features of these pathways.

In this context, multiple prior studies demonstrated HIV-1 gRNAs and/or Gag accumulating at or near the MTOC [75–77]; observations that contrast with our results, and also with recent studies using live cell imaging with subsecond time resolution to demonstrate that gRNA movement in the cytoplasm is predominantly diffusive in nature [71–74], An advantage of our long-term (>24 h) approach is that we observe gRNAs as they first accumulate in the cytoplasm, and prior to (or coincident with) the onset of Gag synthesis. Therefore, we unambiguously demonstrate that Rev/RRE-dependent HIV-1 transcripts flood the cytoplasm with little to no subcellular accumulation at the MTOC or subcellular vesicles (*e*.*g*., Fig 3, left panels and S1 Movie and S6 Movie), in marked contrast to CTE-bearing mRNAs (*e*.*g*., Fig 4). Prior observations of HIV-1 gRNAs at the MTOC or associated with endosomes likely reflect the tendency of Gag/gRNA-enriched virions or assembly intermediates to be re-endocytosed from the plasma membrane at later time points [103,119,120].

We also report for the first time that the RRE regulates *en masse* “burst” gRNA export events wherein, at discrete time points, the proxy MS2-YFP signal is completely re-localized from the nucleus to the cytoplasm in a Rev- and CRM1-dependent fashion (Figs 2, 3 and 8, S1 Movie and S6 Movie). Export-competent heterologous mRNAs bearing an RRE yielded the same phenotype when expressed with Rev (Fig 6A and 6B), thus demonstrating that Rev and the RRE are both necessary and sufficient for this activity. Rev has long been proposed to function as a molecular rheostat, wherein unspliced transcripts in the nucleus await the formation of active Rev/CRM1 export complexes on the RRE [12,121–124]. Consistent with such a model, we observed MS2-YFP punctae to “build up” in the nucleus prior to burst export (Fig 2C and Fig 3D). Such a delay may allow for sufficient production of spliced viral transcripts (*i*.*e*., those encoding Rev, Tat and Nef) prior to the nuclear export of gRNAs and partially spliced transcripts, thereby helping to tune the switch from “early” to “late” viral gene expression.

In infected lymphocytes *in vivo*, such a delay may provide time for the virus to modulate the cell in important ways prior to the onset of virus particle production, for example by degrading CD4 prior to viral Env glycoprotein synthesis because Env mRNAs are partially spliced and thus Rev-dependent [125–127]. Alternatively, exploiting CRM1 (as opposed to NXF1/NXT1) may represent a quicker, more reliable pathway for the virus to ensure late gene expression in times of cell activation and stress. Indeed, there is evidence that T cells preferentially employ CRM1 to mediate nuclear export of select mRNAs transcribed from induced genes as a result of cell activation [128]. Moreover, viral late gene expression in T cells is modulated through G2/M cell cycle arrest induced by the combined activities of the viral accessory factors Vif and Vpr [129,130]. Our studies of gRNA trafficking HeLa cells have been technically successful because they are flat, stationary cells that allow us to integrate single cell measurements from hundreds of cells over 30 hours of continuous imaging, and for several conditions and viruses. However, the need to test the above hypotheses emphasizes the goal of configuring our technology to access and track subcellular dynamics in primary human lymphocytes.

In striking contrast to the Rev/RRE module, we found that CTE-bearing mRNAs and M-PMV genomes were rapidly trafficked to centrosomes soon after leaving the nucleus (Figs 3–8, S2 Movie, S5 Movie and S7 Movie). To our knowledge, this is the first demonstration of a discrete, transferable RNA element capable of linking mRNAs to the microtubule cytoskeleton, and with remarkable specificity. Unlike for HIV-1, there is strong evidence for microtubules and a role for the MTOC in regulating M-PMV capsid assembly and virion egress [95,96,101,102,131]. Regarding the molecular mechanism by which the CTE and NXF1/NXT1 target mRNAs to the MTOC, we found that 1) the 4xCTE was more active than a single CTE in directing heterologous mRNAs to the centrosome (Fig 6D), 2) NXF1 traffics *with* 4xCTE-gRNAs to the MTOC (Fig 4D), 3) that heterologous mRNAs can be readily targeted to the centrosome even in the absence of a CTE by using MS2-NXF1 tethering (Fig 7A–7C), and 4) that a similar result is seen when tethering NXF1 to RRE-gRNAs using Rev-NXF1 fusion proteins co-expressed with NXT1 (Fig 7D–7F). Based on these observations, we propose that cooperative interactions between mRNAs and multiple NXF1/NXT1 molecules serve to signal mRNA engagement of microtubule-based transport machineries. Dynein is the predominant minus-end-directed MT motor protein [132]. Thus, mRNAs bound to NXF1/NXT1 are likely to engage dynein and nucleus-proximal MTs soon after transit across the nuclear pore complex (see model in Fig 9A). Alternatively, NXF1-bound mRNAs can engage centrosomes during metaphase, in conjunction with nuclear membrane breakdown and mitotic spindle formation (see Fig 5 and model in Fig 9B). We have yet to fully interrogate this pathway but note that M-PMV Gag proteins are targeted to the MTOC by an interaction with the dynein-linked adaptor protein Tctex-1 [131]. Moreover, several RNA binding proteins including hnRNPA1 [133], Tpr [134] and the SR protein 9G8 [52] have been implicated in the regulation of M-PMV CTE activity and may affect trafficking linked to cytoplasmic utilization.

That RRE- and CTE-linked gRNA behaviors are different in the cytoplasm is likely to explain, at least in part, prior genetic studies indicating that Rev-dependent and Rev-independent gRNAs are differentially regulated within the cell [30,66,135–137] and associated with inefficiencies to HIV-1 and M-PMV infectious virus propagation [24,55,56]. In this context, we and others have studied a profound block to Gag assembly competency observed in cells derived from mice and other muroid rodents, and rescued by rendering *gag-pol* mRNAs Rev-independent [30,67,138,139]. This defect was recently found to be attributable to a species-specific polymorphism found within muroid rodent (*e*.*g*., mouse and rat) orthologues of CRM1 [140–143] that likely reduces the capacity of Rev/RRE complexes to interface with multiple molecules of CRM1 [144–146]. Thus, similar to CTE activity being a function of NXF1/NXT1 association and abundance (Figs 6D, 7C and 7F), burst export may reflect Rev’s capacity to multimerize on the RRE and recruit a sufficient number of CRM1 molecules. Efforts are ongoing to compare gRNA burst dynamics and cytoplasmic distribution in these permissive versus non-permissive cell systems, with the long-term goal of understanding how to recapitulate these blocks in human cells as a therapeutic strategy.

In summary, we demonstrate contrasting mRNA trafficking behaviors in the cytoplasm programmed by distinct, *cis-*acting retroviral mRNA export elements. Although the bulk of cellular mRNAs are thought to be regulated spatially within the cell [147–149], there are surprisingly few examples of mRNAs restricted to specific subcellular locales in somatic mammalian cells, with the exception of mRNAs encoding actin [150,151] and MT-based axonal transport in neurons [152]. Retroviruses orchestrate complex gRNA cytoplasmic trafficking activities in order to achieve robust cytoplasmic utilization (*i*.*e*., Gag/Gag-Pol translation), and coordinate the convergence of thousands of Gag molecules with a single dimer of gRNAs to form infectious virus particles. The capacity to access and study the integrated stages of mRNA trafficking and protein synthesis using microscopy now allow us to more fully dissect the productive phase of HIV-1 infection and that of other retroviruses, as well as to screen for additional disease-relevant *cis*-acting mRNA elements with roles in trafficking, transcript stability, and protein synthesis.

## Materials and Methods

### Cell culture and plasmids

Human HeLa cervical carcinoma cells and African green monkey Cos7 cells were obtained from the American Type Culture Collection (ATCC), with both cell lines cultured in Dulbecco’s modified Eagle medium (DMEM) supplemented with 10% fetal bovine serum, 1% L-glutamine and 1% penicillin/streptomycin. HeLa.MS2-YFP cells were generated by retroviral transduction. Briefly, *ms2-yfp* cDNAs from pMS2-YFP (a kind gift of Dr. Robert Singer, Albert Einstein School of Medicine, New York, NY) were cloned into the *Bgl*II and *Xho*I cut sites of pCMS28; a MIGRI-derived murine retroviral vector that confers resistance to puromycin [153]. Retroviral vectors were generated as previously described [154] and used to transduce HeLa cells prior to puromycin selection and derivation of a high performance single cell clone using limiting dilution.

The plasmids pcRev, pRev-mCherry, and pRevM10-mCherry were previously described [30,155]. The plasmid encoding mCherry-tubulin [156] was a gift of Dr. Roger Tsien (University of California-San Diego, San Diego, CA). Plasmids encoding MS2-Rev, MS2-CRM1, and MS2-NXF1 [90,91] were a gift of Dr. Bryan Cullen (Duke University, Durham, NC). The plasmid encoding NXT1 [50] was a gift of Drs. Marie Hammarsjköld and David Rekosh (University of Virginia, Charlottesville, VA). Base plasmids encoding RRE-bearing surrogate HIV-1 gRNAs expressing Gag-CFP were generated by; 1) inserting unique *Sac*II and *BsmB*I restriction cut sites between the *gag* and *pol* reading frames of a pcDNA3.1-based plasmid pGag-Pol-Vif-RRE [53]; 2) inserting a cassette encoding twenty-four copies of MS2 binding loops (24xMBL) using *Sac*II and *Bam*HI sticky ends; and 3) replacing the native *gag* reading frame with a *gag* cDNA fused in frame to *cfp* and separated by sequence encoding a glycine-rich linker (PGISGGGGGILD) using *Sac*I and *Sac*II cut sites. pGag-Pol-Vif-RRE encodes the first 5,297 nucleotides of the HIV-1_NL4-3_ RNA genome under the transcriptional control of the cytomegalovirus immediate early (CMV-IE) promoter, and upstream of an HIV-1_IIIB_-derived RRE (HIV-1_IIIB_ nts 7708–8058) and polyadenylation signal. To generate 1xCTE- and 4xCTE gRNA constructs, the CTE from M-PMV (nucleotides 8007–8176) or multimeric (4X) CTE [29] were used to replace the RRE in pRRE-gRNA using *EcoR*I-*Stu*I fragments derived from 1xCTE- and 4xCTE-pGag-Pol-Vif constructs [30]. CFP-MSL plasmids (Fig 6) were generated by inserting *cfp* cDNAs into pcDNA3.1 using *Nhe*I and *Bam*HI cut sites, adding the 24xMBL cassette described above using *Bam*HI and *Not*I, and then inserting export element sequences using either *Stu*I-*Xho*I (RRE) or *Dra*I-*Xho*I (1xCTE and 4xCTE) cut fragments taken from the Gag-CFP/24xMBL constructs described above. Full-length Gag-CFP/24xMBL HIV-1 gRNA constructs were generated by transferring a *Spe*I-*Age*I fragment from pRRE-gRNA into pNL4-3.Luc.R-E-, a plasmid encoding full-length HIV-1_NL4-3_ mutated to not express the viral Envelope (E-) and Vpr (R-) proteins, with the firefly *luciferase* reading frame inserted into the position of the viral *nef* gene. pNL4-3.Luc.R^–^.E^−^was obtained through the NIH AIDS Reagent Program, Division of AIDS, NIAID, NIH: from Dr. Nathaniel Landau [93,94]. Full-length Gag-CFP/24xMBL M-PMV was generated by replacing the Gag-GFP reading frame in plasmid pSARM-GagGFP-M100 [96] (a kind gift of Eric Hunter, Emory University) with Gag-CFP cDNA using overlapping PCR and *Mlu*I-*Nhe*I cut sites, prior to inserting the 24xMBL cassette at the end of the *gag* reading frame into engineered *Sac*I *and Bst*BI sites. Plasmids encoding Rev- and RevM10-NXF1 fusion proteins were generated by fusing *rev* or *revM10* reading frames with that of *nxf1* separated by a glycine-rich linker using overlapping PCR, and subsequently cloned into pcDNA3.1 using *Eco*RI and *Not*I restriction cut sites. All plasmids were verified using restriction digest and DNA sequencing.

### Virus particle assembly assays

HeLa.MS2-YFP cells were plated at 30% confluency in 6-well tissue culture treated dishes and then transfected using FuGENE 6 reagent (Promega) as per the manufacturer’s instructions. Transfection mixes included 1000 ng of gRNA plasmids mixed with 500 ng of plasmids encoding Rev-mCherry or a mCherry control. The cell medium was replaced at 24 h post-transfection, and cell lysates and supernatants were harvested at 48 h prior to immunoblot as previously described [142] using mouse anti-p24^Gag^ (24–2) [157], mouse anti-p24^Gag^ (derived from hybridoma 183-H12-5C developed by Dr. Bruce Chesebro and obtained from the NIH AIDS Reagent Program, Division of AIDS, NIAID, NIH) [158], or rabbit anti-Hsp90 (Santa Cruz Biotechnology) antisera prior to incubation with the appropriate anti-mouse or anti-rabbit secondary antibodies conjugated to the infrared fluorophores IRDye800 or IRDye680 (Li-Cor Biosciences).

### Immunofluorescence and fixed cell analyses

For immunofluorescence-based detection of Gag, Rev, Pericentrin, or NXF1, HeLa or HeLa.MS2-YFP cells were plated on glass coverslips, transfected as described above, and then fixed at 20–36 hours post-transfection in 4% paraformaldehyde prior to cell permeabilization using 0.1% Triton-X-100 and then 0.02% SDS for 10 min at room temperature. Primary antibodies specific for Gag (24–2) [157], Rev (Rev-6) [155], Pericentrin (Abcam, ab4448) or NXF1 (Thermo Scientific, PA5-21415) were added for one hour prior to washing and detection using secondary antibodies conjugated to AlexaFluor594 (Invitrogen). Fluorescence *in situ* hybridization (FISH) was performed using digoxigenin-labeled RNA probes specific to *gag-pol* nucleotides 1353–1589 and were prepared according to the manufacturer’s instructions (Roche Diagnostics). For quantitative analysis of fluorescently-tagged gRNA, Rev, and Gag subcellular localization (Figs 1D and 6B and S2 Fig), cells were fixed as above at 36 h post-transfection, with images acquired for CFP, YFP and mCherry channels for >500 cells using a 10X (0.5 N.A.) objective lens prior to scoring at least 100 mCherry- or Rev-mCherry-expressing cells for the relative localization of each factor. Experiments were carried out in triplicate, with samples coded prior to image acquisition and scored by a blind observer. For quantification of gRNA targeting to the MTOC (Figs 4E and 6D), cells were transfected to express RRE-gRNAs and Rev, or 4xCTE-gRNAs with or without the addition of 3 μM nocodazole (Sigma) prior to fixation at 20 h post-transfection and scoring for MS2-YFP signal clustered near centrosomes as indicated by Pericentrin immunostaining, as described above.

### Live cell microscopy and analysis

For all time-lapse imaging experiments, cells were plated in 8-well No. 1.5H glass bottom slides (Ibidi) maintained at 37°C, ~50% humidity and 5% CO_2_ in a Pathology Devices LIVECELL incubator (Pathology Devices, Inc.). To label cell nuclei, cells were incubated with Hoeschst 33342 NucBlue Live Cell Stain ReadyProbes (Life Technologies) according to the manufacturer’s instructions. Imaging experiments were performed on a Nikon Ti-Eclipse inverted wide-field epifluorescent deconvolution microscope (Nikon Corporation) or a similarly equipped Nikon Ti-Eclipse confocal microscope (Bruker). Imaging was carried out for 16–48 h starting at approximately 6 h post-transfection. Long-term movies were collected using either a CoolSnap HQ (Photometrics) or Orca-Flash 4.0 C11440 (Hamamatsu) camera and Nikon NIS Elements software (v 4.00.03) using 10x (N.A. 0.5), 20x (N.A. 0.75) or 40x oil (N.A. 1.3) objective lenses. Single images were typically acquired either every 30 or 60 minutes using the following excitation/emission filter sets (wavelengths in nm): 325-375/435-485 (Hoeschst), 418-442/458-482 (CFP), 490-520/520-550 (YFP), and 565-590/590-650 (mCherry). For movies with subsecond time resolution, gRNA punctae were tracked using a 100x (N.A. 1.45) oil objective lens with images collected every 700 ms using the YFP filter set. All movies were post-processed and analyzed using either NIS Elements software or FIJI/ImageJ2 [159,160] plugins integrated into the Konstanz Information Miner (KNIME) image analysis platform [161].

For the single cell viral protein and gRNA expression analysis presented S1 Fig and S2 Fig, Gag-CFP, Rev-mCherry, or MS2-YFP gRNA channels were collected every hour for 24–36 hours using a 20X objective lens. Background fluorescence was measured adjacent to selected cells and subtracted prior to quantification of mean fluorescence intensity (MFI) for the indicated cellular compartments. NIS elements was used to carefully draw nuclear and cytoplasmic regions of interest (ROI) for each cell beginning at the first frame instance that Rev-mCherry expression was detected above background (or CFP for S2 Fig panel B). Cytoplasmic MFI was recorded over time for Rev-mCherry or Gag-CFP, and nuclear and cytoplasmic MFI were recorded for the MS2-YFP gRNA signal in order to derive a cytoplasmic to nucleus (C:N) ratio demonstrating transitions of MS2-YFP from the nucleus to the cytoplasm. The range of MFI values or C:N ratios over time was normalized to its respective minimum and maximum value for each cell. Cell measurements were aligned to the minimum Rev value, and the average normalized intensity value (n = 30) for each time point was calculated and plotted for individual cell measurements. In Fig 2B, the normalized peak C:N ratio for cells was identified (value of 1) and all cells were aligned relative to the maximal expression value. This value is thus representative of the peak nuclear export “burst” when the MS2-YFP signal in the cytoplasm is greatest compared to the nucleus.

## Supporting Information

S1 Fig Expression kinetics for 3-color HIV-1 viruses over the entire productive phase. RRE-gRNAs encoding Gag-CFP and Rev-mCherry were tracked in 30 cells for 25 hours. **(A)** Increases to Rev-mCherry in the cytoplasm over time over 25 hours, normalized to the maximum and minimum mean fluorescence intensity (MFI). **(B)** As for (A), but tracking Gag-CFP from the time of first detection of Rev-mCherry (t = 0). **(C)** MS2-YFP (RRE-gRNA) cytoplasmic to nuclear (C:N) ratios of MFI over time. **(D)** Plot combining normalized MFI rise kinetics for Gag-CFP and Rev-mCherry (left y-axis) and changes to C:N ratio for MS2-YFP (RRE-gRNA) (right y-axis).
(TIF)

S2 Fig RRE- vs. CTE-dependent effects on HIV-1 gRNA and Gag subcellular distribution and abundance. **(A)** HeLa.MS2-YFP cells were transfected with the indicated constructs and then fixed at 36 h post-transfection. Phenotypes were scored as for Fig 1E. Images on right depict typical “Nuclear” and “Nuclear + Cytoplasmic” phenotypes observed for the 4xCTE-gRNA condition. “N” indicates the nucleus and “C” indicates the cytoplasm. Note that the 1xCTE-gRNA condition yielded very little MS2-YFP signal in the cytoplasm (middle graph) and, accordingly, only low levels of Gag-CFP expression (lower graph). (**B)** Plot comparing normalized MFI cytoplasmic rise kinetics for Gag-CFP derived from either RRE-gRNA vs. 4xCTE-gRNA transcripts, or a CFP control. Solid line represents the average and background trace the standard deviation for >50 cells per condition. **(C)**. Representative images from an experiment as for S2 Fig panel A showing Gag-CFP and gRNA distributions for the RRE-gRNA and 4xCTE-gRNA conditions. Arrow points to Gag-CFP co-localizing with 4xCTE-gRNAs in a perinuclear region.
(TIF)

S1 Movie 3-color imaging of HIV-1 Rev/RRE-dependent gRNA trafficking and virion assembly. Time lapse imaging of RRE-gRNAs (MS2-YFP) co-expressed with Rev-mCherry and expressing Gag-CFP in HeLa.MS2-YFP cells. Cells were imaged starting at 6 h post-transfection with images acquired every 2.5 hours for greater than 17.5 h with the final frame acquired at 21 h. Movie corresponds to Fig 3D.
(AVI)

S2 Movie Live cell imaging reveals 4xCTE-gRNAs coalescing at a perinuclear region. Time lapse imaging of 4xCTE-gRNAs (MS2-YFP, green) expressing Gag-CFP (blue) in HeLa.MS2-YFP cells. Cells were imaged starting at 6 h post-transfection with images acquired every 2.5 hours for 27.5 h. Movie corresponds to Fig 3E. Arrows indicate nuclear punctae clustering over time in the cytoplasm but near the nuclear membrane.
(AVI)

S3 Movie Bi-directional 4xCTE-gRNA trafficking near the centrosome. Time lapse imaging of 4xCTE-gRNAs (MS2-YFP, green) co-expressed with mCherry-Tubulin (red) in a HeLa.MS2-YFP cell. Cell was imaged starting at 24 h post-transfection with images acquired every 500 ms for 10 s. Arrows indicate 4xCTE-gRNAs moving bidirectionally on a microtubule near the centrosome.
(AVI)

S4 Movie Bi-directional CTE-gRNA trafficking in the cytoplasm. Time lapse imaging of 4xCTE-gRNAs (MS2-YFP, black signal) in the cytoplasm of a HeLa.MS2-YFP cell. Cell was imaged starting at 24 h post-transfection with images acquired at ~0.7 s intervals for 57 s. Movie corresponds to Fig 4C. Colored tracks indicate 4xCTE-gRNAs moving in rectilinear paths in the cytoplasm both towards and away from the MTOC.
(AVI)

S5 Movie 4xCTE-gRNAs are targeted to centrosomes during cell division. Time lapse imaging of CTE-gRNAs (MS2-YFP, green), mCherry-Tubulin (red) and DNA (Hoescht, blue) in HeLa.MS2-YFP cells. Cells were imaged starting at 6 h post-transfection with images acquired at up to 1 hr intervals for ~21 h. Movie corresponds to Fig 5A.
(AVI)

S6 Movie Burst nuclear export and translation of full-length HIV-1 gRNAs. Time lapse imaging of Gag-CFP/24xMBL HIV-1 gRNAs (MS2-YFP channel, green) and Gag-CFP (blue) in HeLa.MS2-YFP cells. MS2-YFP transitioned from the nucleus to the cytoplasm over 27 hours with Gag-CFP expression initiated during build-up of MS2-YFP signal in the cytoplasm.
(AVI)

S7 Movie M-PMV-gRNAs trafficking from nucleus to MTOC. Time lapse imaging of M-PMV-gRNAs (MS2-YFP) trafficking in the cytoplasm of HeLa.MS2-YFP cells. Red arrows indicate movement of a representative MS2-YFP punctae (M-PMV gRNA) moving between the nuclear membrane and the centrosome. Images were acquired every ~0.7 s for 28 s. Black arrows indicate the MTOC.
(AVI)
