# Supplementary figures and images for: HIV-1 and M-PMV RNA Nuclear Export Elements Program Viral Genomes for Distinct Cytoplasmic Trafficking Behaviors
Source: PLoS Pathog. 2016 Apr 12;12(4):e1005565. doi: 10.1371/journal.ppat.1005565 (PMC4829213; doi:10.1371/journal.ppat.1005565)

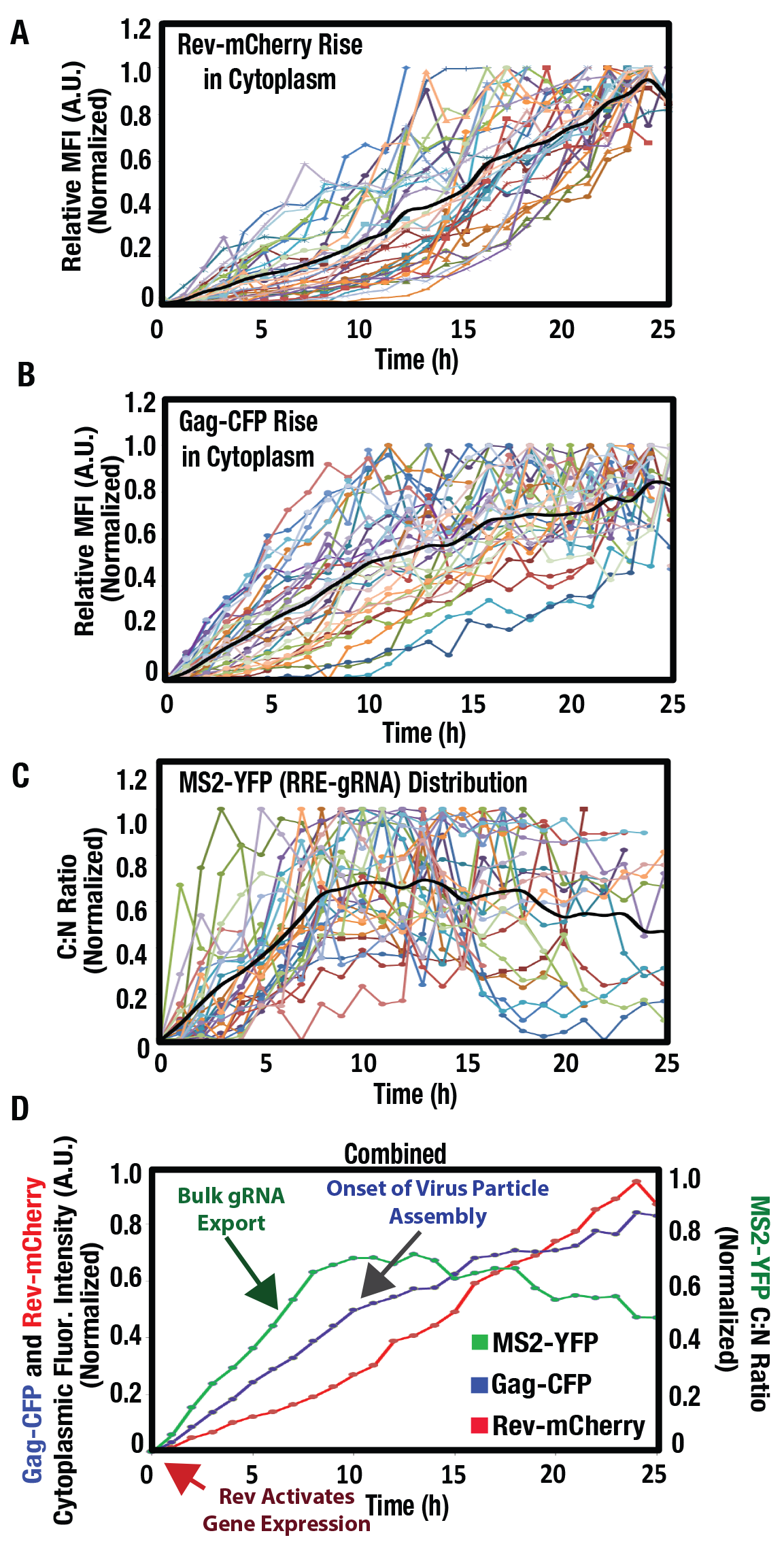

Supplement: S1 Fig — RRE-gRNAs encoding Gag-CFP and Rev-mCherry were tracked in 30 cells for 25 hours. (A) Increases to Rev-mCherry in the cytoplasm over time over 25 hours, normalized to the maximum and minimum mean fluorescence intensity (MFI). (B) As for (A), but tracking Gag-CFP from the time of first detection of Rev-mCherry (t = 0). (C) MS2-YFP (RRE-gRNA) cytoplasmic to nuclear (C:N) ratios of MFI over time. (D) Plot combining normalized MFI rise kinetics for Gag-CFP and Rev-mCherry (left y-axis) and changes to C:N ratio for MS2-YFP (RRE-gRNA) (right y-axis). (TIF) [file ppat.1005565.s001.tif]

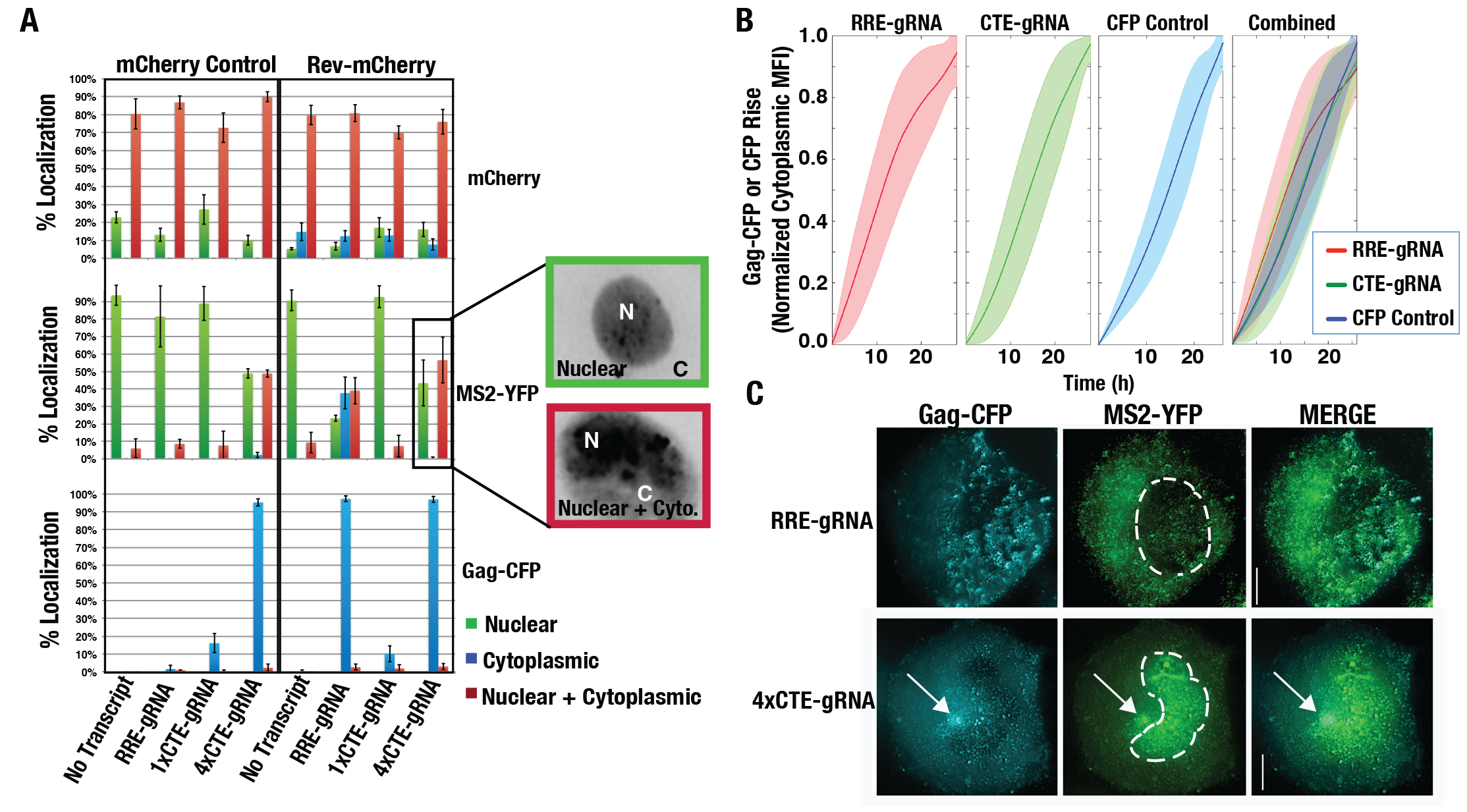

Supplement: S2 Fig — (A) HeLa.MS2-YFP cells were transfected with the indicated constructs and then fixed at 36 h post-transfection. Phenotypes were scored as for Fig 1E. Images on right depict typical “Nuclear” and “Nuclear + Cytoplasmic” phenotypes observed for the 4xCTE-gRNA condition. “N” indicates the nucleus and “C” indicates the cytoplasm. Note that the 1xCTE-gRNA condition yielded very little MS2-YFP signal in the cytoplasm (middle graph) and, accordingly, only low levels of Gag-CFP expression (lower graph). (B) Plot comparing normalized MFI cytoplasmic rise kinetics for Gag-CFP derived from either RRE-gRNA vs. 4xCTE-gRNA transcripts, or a CFP control. Solid line represents the average and background trace the standard deviation for >50 cells per condition. (C). Representative images from an experiment as for S2 Fig panel A showing Gag-CFP and gRNA distributions for the RRE-gRNA and 4xCTE-gRNA conditions. Arrow points to Gag-CFP co-localizing with 4xCTE-gRNAs in a perinuclear region. (TIF) [file ppat.1005565.s002.tif]
